# Modulation of Synaptic Plasticity by Glutamatergic Gliotransmission: A Modeling Study

**DOI:** 10.1155/2016/7607924

**Published:** 2016-04-18

**Authors:** Maurizio De Pittà, Nicolas Brunel

**Affiliations:** ^1^Department of Neurobiology, The University of Chicago, Chicago, IL 60637, USA; ^2^Project-Team BEAGLE, INRIA Rhône-Alpes, 60097 Villeurbanne, France; ^3^Departments of Statistics and Neurobiology, The University of Chicago, Chicago, IL 60637, USA

## Abstract

Glutamatergic gliotransmission, that is, the release of glutamate from perisynaptic astrocyte processes in an activity-dependent manner, has emerged as a potentially crucial signaling pathway for regulation of synaptic plasticity, yet its modes of expression and function in vivo remain unclear. Here, we focus on two experimentally well-identified gliotransmitter pathways, (i) modulations of synaptic release and (ii) postsynaptic slow inward currents mediated by glutamate released from astrocytes, and investigate their possible functional relevance on synaptic plasticity in a biophysical model of an astrocyte-regulated synapse. Our model predicts that both pathways could profoundly affect both short- and long-term plasticity. In particular, activity-dependent glutamate release from astrocytes could dramatically change spike-timing-dependent plasticity, turning potentiation into depression (and vice versa) for the same induction protocol.

## 1. Introduction

In recent years, astrocytes have attracted great interest for their capacity to release neuroactive molecules, among which are neurotransmitters like glutamate, because these molecules could modulate neural activity and lead to a possible role for astrocytes in neural information processing [1–3]. Indeed, astrocyte-derived neurotransmitters, also called “gliotransmitters” for their astrocytic origin [4], have been shown to act on neurons and to regulate synaptic transmission and plasticity through a variety of mechanisms [5]. The binding of receptors located on either pre- or postsynaptic terminals by astrocyte-released glutamate has historically been the first pathway for gliotransmission to be discovered and, arguably, the most studied one experimentally for its several possible functional implications [6].

Activation of extrasynaptic receptors on presynaptic terminals by astrocytic glutamate modulates the probability of neurotransmitter release from those terminals [6]. In particular, depending on receptor type, such modulation may be either toward an increase or toward a decrease of the frequency of spontaneous [7–11] and evoked neurotransmitter release in both excitatory [2, 8, 10, 12] and inhibitory synapses [13–15]. Because synaptic release probability characterizes how a synapse filters or, in other words, “processes” presynaptic action potentials [16, 17], modulations of synaptic release probability by astrocytic glutamate are suggested to alter the computational properties of neural circuits [18].

Glutamate released by astrocytes may also bind to extrasynaptically located postsynaptic NMDA receptors, evoking slow inward currents (SICs) in nearby neurons [11, 19–26]. The depolarizing action of these currents modulates neural excitability with the potential to affect neuronal action potential firing [27]. Moreover, because single astrocytes are in close proximity to a large number (~100) of neurons [28], it has been suggested that an inward current can be generated in many adjacent neurons, thereby promoting synchrony of neuronal firing [19–21].

Although modulations of both synaptic release and SICs mediated by glutamatergic gliotransmission have been recorded in the cortex and the hippocampus, as well as in several other brain regions [5], their physiological relevance remains elusive. In particular, beyond regulation of synaptic filtering and neuronal firing, theoretical arguments support a further possible role for both pathways in the regulation of NMDAR-mediated spike-timing-dependent plasticity (STDP) [29]. Both pathways have the potential to regulate activation of postsynaptic NMDA receptors and, in doing so, glutamatergic gliotransmission could ultimately regulate the STDP outcome, that is, either potentiation (LTP) or depression (LTD) [30, 31]. Consistent with this hypothesis, experiments have reported a lower threshold for LTP induction at hippocampal synapses when synaptic release is increased by astrocytic glutamate [9]. Moreover, long-term potentiation responses of neurons in the primary visual cortex by cholinergic activation of surrounding astrocytes has also been reported to be correlated with an increase of SIC frequency in those neurons [32].

While the potential impact on STDP of pre- or postsynaptic activity-dependent modulations of synaptic efficacy has widely been addressed both experimentally [33] and theoretically [34, 35], the possible effect on plasticity of the regulation of these modulations by glutamatergic gliotransmission (and by gliotransmission in general) has been investigated by very few theoretical studies. These studies suggest a potential role in LTP induction both for large increases of synaptic release and for large SICs mediated by astrocytic glutamate [36, 37]. This scenario seems however at odds with the majority of recent experimental observations that report modest signaling magnitudes for these two routes of gliotransmission. It is thus not clear under what biophysical conditions modulations of synaptic release or SICs mediated by glutamatergic gliotransmission could affect STDP. Astrocyte-mediated SICs, for example, are known to occur sporadically, being recorded in single neurons only as often as <5/min [26, 32], raising the question of whether and how, by occurring at such low rates, they could effectively play a role in STDP.

We thus set to investigating what conditions are required for glutamatergic gliotransmission to affect STDP by presynaptic modulations of neurotransmitter release or through postsynaptic SICs. We extend the model of an astrocyte-regulated synapse originally introduced by De Pittà et al. [73] to include a biophysically realistic description of synaptically evoked gliotransmitter release by the astrocyte as well as a mechanism for the generation of postsynaptic SICs and STDP. Extensive numerical investigations of our model lead to two major predictions. First, glutamatergic gliotransmission could change the nature of STDP by modifying the parameter ranges for LTP and LTD induction. Second, this effect crucially depends on the nature of gliotransmission, that is, whether it is release-increasing or release-decreasing, its strength, and its rate of occurrence and when it occurs with respect to pre/post pairs. Thus, while glutamatergic gliotransmission could potentially play a role in STDP and learning, in practice this effect must satisfy several biophysical and activity-dependent constraints, supporting the existence of specialized dynamic interactions between astrocytes and neurons.

## 2. Biophysical Modeling of a Gliotransmitter-Regulated Synapse

**Figure 1:**
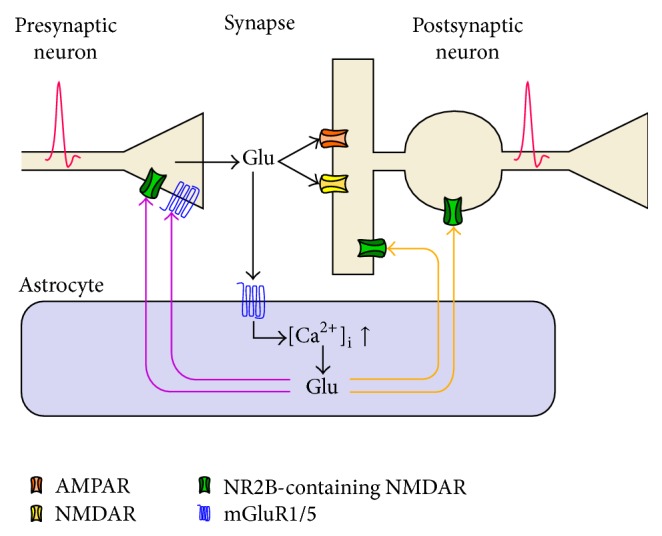
Pathways of glutamatergic gliotransmission. Perisynaptic astrocytic processes in several brain areas and different excitatory (but also inhibitory) synapses may release glutamate in a Ca^2+^-dependent fashion. In turn, released astrocytic glutamate may increase (or decrease) synaptic neurotransmitter release by activating extrasynaptically located presynaptic receptors (*magenta arrows*) or contribute to postsynaptic neuronal depolarization by binding to extrasynaptic NMDA receptors (*orange arrows*) which mediate slow inward currents (SICs). These receptors often (but not always) contain NR2B subunits and are thus different with respect to postsynaptic NMDARs. Glutamate release by the astrocyte could be triggered either by activity from the same synapses that are regulated by the astrocyte (homosynaptic scenario) or by other synapses that are not directly reached by glutamatergic gliotransmission (heterosynaptic scenario).

Although there may be several possible routes by which astrocytes release glutamate [38–40], Ca^2+^-dependent glutamate release is likely the main one in physiological conditions [41, 42]. From a modeling perspective, as illustrated in Figure 1, Ca^2+^-dependent glutamatergic gliotransmission consists of three distinct signaling pathways. One pathway (*black arrows*) initiates the release-triggering Ca^2+^ signal in the astrocyte and may be either exogenous or heterosynaptic or be triggered by the very synapses that are modulated by glutamatergic gliotransmission in a homosynaptic fashion. The other two pathways are instead represented by the two recognized routes for the action of glutamatergic gliotransmission on synaptic terminals: the presynaptic pathway whereby astrocytic glutamate modulates synaptic release (*magenta arrows*) and the postsynaptic pathway which mediates SICs in nearby neurons (*orange arrows*). Although both pathways could coexist at the same synapse in principle [11], their functional regulation is probably through different Ca^2+^-dependent pathways [26], both in terms of spatiotemporal Ca^2+^-dynamics [24] and in terms of pools of releasable glutamate resources and/or mechanism of release for these latter [43]. Thus, in the following, we set to investigating the effect of synaptic transmission of each pathway independently of the other.

### 2.1. Calcium-Dependent Gliotransmitter Release

We begin our study by a description of a biophysically realistic model of synaptically evoked Ca^2+^-dependent glutamate release from an astrocyte. At excitatory [44] and inhibitory synapses [45], astrocytes can respond to synaptically released neurotransmitters by intracellular Ca^2+^ elevations and release glutamate in turn [6]. Although morphological and functional details of the coupling between synaptic terminals and the surrounding astrocytic processes remain to be fully elucidated, the current hypothesis is that synaptically evoked glutamate-releasing astrocytic Ca^2+^ signaling is mainly by spillover of synaptic neurotransmitters and/or other factors, which bind to high-affinity astrocytic G protein-coupled receptors (GPCRs) [5] and thereby trigger inositol 1,4,5-trisphosphate (IP_3_) production and Ca^2+^ release from the endoplasmic reticulum (ER) [46–48]. While early work mainly monitored somatic Ca^2+^ increases concluding that astrocytes respond only to intense neuronal firing patterns [49], recent experiments in astrocytic processes revealed that astrocytes may also respond to low levels of synaptic activity by Ca^2+^ elevations confined in subcellular regions of their processes [10, 48, 50], suggesting that the profile of astrocytic Ca^2+^ signaling and thus glutamate release could span the whole spectrum of neuronal (synaptic) activity [5].

To realistically describe synaptic release in the whole spectrum of neuronal firing, we consider the model of an activity-dependent synapse first introduced by Tsodyks and Markram [51]. This model captures the dependence of synaptic release on past activity, that is, presynaptic short-term plasticity, which substantially influences synaptic transmission at high enough rates of neuronal firing [52]. In particular, synaptic release results from the product of two quantities: (i) the probability of neurotransmiter-containing vesicles to be available for release and (ii) the probability of such vesicles to be effectively released by an action potential [53], which correlates with intrasynaptic Ca^2+^ [54]. At rest, it is assumed that all vesicles are available for release. The arrival of an action potential opens presynaptic voltage-dependent Ca^2+^ channels that trigger a transient increase of intrasynaptic Ca^2+^ which promotes release of a fraction *u*_*S*_ of available vesicles. Following release, the emptied vesicles are refilled in some characteristic time *τ*_*d*_, while intrasynaptic Ca^2+^ and thus vesicle release probability decay to zero with a different time constant *τ*_*f*_. For multiple action potentials incoming at time intervals of the order of these two time constants, neither vesicle replenishment nor intrasynaptic Ca^2+^ are restored to their resting values, so that the resulting synaptic release depends on the history of synaptic activity [55].

**Figure 2:**
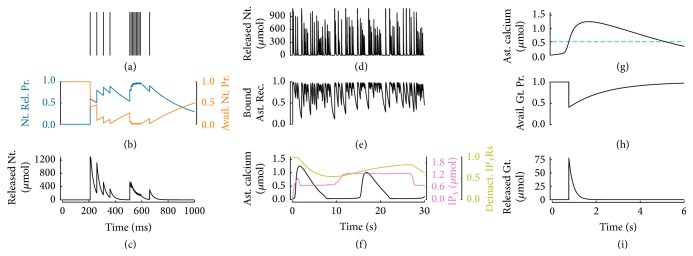
Biophysical modeling of a gliotransmitter-regulated synapse. ((a)–(c)) Model of synaptic release. Incoming presynaptic spikes (a) increase intrasynaptic Ca^2+^ levels which directly control the probability of release of available neurotransmitter resources ((b), Nt. Rel. Pr.) and decrease, upon release, the fraction (or probability) of neurotransmitter-containing vesicles available for release (Avail. Nt. Pr.). Each spike results in release of a quantum of neurotransmitter from the synapse ((c), Released Nt.) whose concentration in the perisynaptic space decays exponentially. Synapse parameters: *τ*_*d*_ = 0.5 s, *τ*_*f*_ = 0.3 s, and *U*_0_ = 0.6. Stimulation by Poisson-distributed APs with an average rate of 5 Hz. ((d)–(f)) Model for astrocyte activation. Synaptically released neurotransmitter in the perisynaptic space (d) binds astrocytic receptors ((e), Bound Ast. Rec.), resulting in IP_3_ production which triggers Ca^2+^ signaling in the astrocyte (f). This latter also depends on the fraction of deinactivated IP_3_ receptors/Ca^2+^ channels (Deinact. IP_3_Rs) on the astrocyte ER membrane (see Appendix A.1). ((g)–(i)) Model for gliotransmitter release. The increase of astrocytic Ca^2+^ beyond a threshold concentration ((g),* cyan dashed line*) results in the release of a quantum of gliotransmitter, which decreases the probability of further release of gliotransmitter ((h), Avail. Gt. Pr.) while transiently increasing extracellular gliotransmitter concentration ((i), Released Gt.). Model parameters as in Table 1.

We illustrate the response of the synapse model to a train of action potentials in Figures 2(a)–2(c). The low rate of stimulation of the first four action potentials (Figure 2(a)) allows for the reintegration of most of the released neurotransmitter in between action potentials thereby keeping vesicle depletion limited (Figure 2(b),* orange trace*). In parallel, intrasynaptic Ca^2+^ grows, and so does vesicle release probability (Figure 2(b),* blue trace*), resulting in progressively larger release of neurotransmitter per action potential or, in other words, in short-term facilitation of synaptic release (Figure 2(c), *t* < 500 ms). On the contrary, the presentation of a series of action potentials in rapid succession at *t* = 500 ms results in a sharp increase of vesicle release probability to a value close to saturation (i.e., Nt. Rel. Pr.≃1) which causes exhaustion of neurotransmitter resources (i.e., Avail. Nt. Pr.≃0). In this scenario, therefore, from one spike to the next one, progressively fewer neurotransmitter resources are available for release and the amount of released resources decreases with incoming action potentials, leading to depression of synaptic transmission. Such depression is short-lived, since synaptic release tends to recover after a sufficiently long period in which no action potentials occur, that is, the case, for example, of the last action potential at *t* = 800 ms.

Once released into the synaptic cleft, synaptic neurotransmitter is rapidly cleared by diffusion as well as by other mechanisms, including uptake by transporters and/or enzymatic degradation [56, 57]. In the simplest approximation, the contribution of these mechanisms can be modeled by a first-order reaction [58] which accounts for the exponentially decaying profile of neurotransmitter concentration in Figure 2(c) after synaptic release at each action potential. A fraction of released neurotransmitter molecules also spills out of the synaptic cleft to the perisynaptic space (Figure 2(d)) where it binds to GPCRs on the astrocyte (Figure 2(e)), therein triggering Ca^2+^ signaling (Figure 2(f)). To quantitatively describe this process, we modify the model of GPCR-mediated Ca^2+^ signaling originally introduced by De Pittà et al. [141] to account for dynamic regulation of astrocytic receptors by synaptic activity (see Appendix A, Section A.1). Accordingly, as illustrated in Figure 2(f), GPCR-mediated Ca^2+^ signaling is a result of the nonlinear interplay of three processes: (i) IP_3_ production by GPCRs bound by synaptic neurotransmitter (*magenta trace*), (ii) Ca^2+^ release from the ER into the cytosol, which is triggered by IP_3_-bound Ca^2+^ channels (IP_3_Rs) and also modulates cytosolic IP_3_ (*black trace*), and (iii) the effective fraction of available or, more exactly, “deinactivated” IP_3_Rs [59] that can take part in Ca^2+^ release from the ER (*yellow trace*). Depending on the choice of parameter values, the astrocyte model may display both large, long-lasting somatic Ca^2+^ elevations and smaller and shorter Ca^2+^ increases, akin to those reported in astrocytic processes [47] (see Appendix B).

Glutamate release from the astrocyte is then assumed to occur every time that Ca^2+^ increases beyond a threshold concentration (Figure 2(g),* cyan dotted line*), in agreement with experimental observations [60, 61]. Although different mechanisms for glutamate release by the astrocyte could be possible, a large amount of evidence points to vesicular exocytosis as the main one to likely occur on a physiological basis [62]. Because astrocytic glutamate exocytosis bears several similarities with its synaptic homologue (reviewed in De Pittà et al. [29]), we model it in the same fashion. Thus, in line with experimental observations [63, 64], we postulate the existence of an astrocytic vesicular compartment that is competent for regulated glutamate exocytosis. Then, upon a suprathreshold Ca^2+^ elevation, a fixed fraction of astrocytic glutamate-containing vesicles releases glutamate into the extracellular space. Glutamate is then reintegrated into the astrocyte with some characteristic time constant (Figure 2(h)). In this fashion, glutamate concentration in the extracellular space abruptly increases by exocytosis from the astrocyte and then exponentially decays akin to neurotransmitter concentration in the synaptic cleft, yet, in general, at a different rate (Figure 2(h)) (Appendix B).

The description of gliotransmitter release hitherto introduced ignores the possible stochastic nature of astrocytic glutamate release [65] and reproduces the total amount of glutamate released, on* average*, by a single Ca^2+^ elevation beyond the release threshold. This description provides a simplified general framework to realistically capture synaptically evoked glutamate release by the astrocyte independently of the underlying mechanism of astrocytic exocytosis, which may either be in the form of a burst of synchronous vesicle fusion events that peaks within the first 50–500 ms from the Ca^2+^ rise underneath the plasma membrane [61, 65, 66] or occur at slower fusion rates in an asynchronous fashion [67, 68].

### 2.2. Gliotransmitter-Mediated Regulation of Synaptic Release and Short-Term Synaptic Plasticity

Once released, astrocyte-derived glutamate can diffuse in the extracellular space and bind extrasynaptic receptors located on presynaptic terminals. In particular, ultrastructural evidence suggests colocalization of glutamate-containing vesicles in perisynaptic astrocytic processes with those receptors [8], hinting a focal action of astrocytic glutamate on these latter. Such action is likely spatially confined and temporally precise, akin to that of a neurotransmitter on postsynaptic receptors, and is not affected by synaptic neurotransmitters [6]. Both ionotropic and metabotropic presynaptic receptors may be activated by astrocytic glutamate, yet their differential recruitment likely depends on developmental, regional, physiological, and cellular (synaptic) factors (reviewed in [29]). The details of the biochemical mechanisms of action of these receptors on synaptic physiology are not fully understood [69], but the simplest explanation is that they all modulate intrasynaptic Ca^2+^ levels, eventually increasing or decreasing synaptic release probability [18], although in a receptor-specific fashion [52, 69, 70].

**Figure 3:**
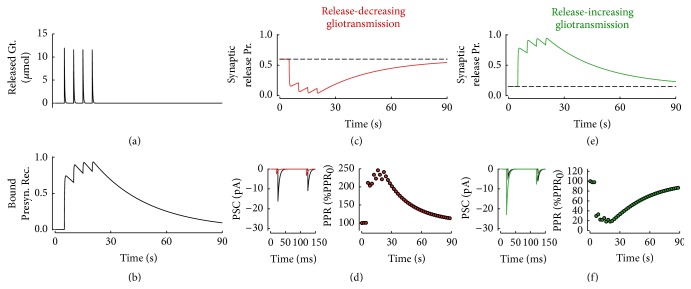
Presynaptic pathway of gliotransmission. Gliotransmitter released from the astrocyte (a) binds extrasynaptically located presynaptic receptors (b) thereby decreasing or increasing synaptic release depending on the type of gliotransmitter and receptor. In the release-decreasing case, synaptic release probability could approach zero by gliotransmission ((c),* red trace*, *ξ* = 0), although, in practice, less dramatic reductions are more likely to be measured with respect to the original value in the absence of gliotransmission (*black dashed line*). The reduction in synaptic release probability changes pair pulse plasticity increasing the pair pulse ratio (d). In the case of release-increasing gliotransmission, synaptic release probability could instead increase up to one ((e),* green trace*, *ξ* = 1). In turn, pair pulse plasticity changes towards a decrease of the ensuing pair pulse ratio (f). Parameters as in Table 1 except for *ϱ*_*e*_ = 10^−4^, *O*_*P*_ = 0.6 *μ*M^−1^ s^−1^, *τ*_*P*_ = 30 s, *ζ* = 0.54, *J*_*S*_ = 3 mV, and *R*_in_ = 60 M*Ω*.

From a modeling perspective, as originally proposed by De Pittà et al. [73], the common effect on synaptic release shared by different receptors allows expressing, in the simplest approximation, the synapse's resting release probability proportionally to the fraction of presynaptic receptors activated by astrocytic glutamate (Appendix A, Section A.1). In this fashion, as illustrated in Figure 3, the time evolution of the fraction of activated presynaptic receptors ensuing from a series of glutamate release events by the astrocyte (Figures 3(a) and 3(b)) is reflected by the dynamics of synaptic release probability at rest averaged across different trials (Figures 3(c) and 3(e)). The value of the coefficient of proportionality for the dependence of synaptic release probability on receptor activation sets the type of modulation of synaptic release by astrocytic glutamate which can be either release-decreasing (Figure 3(c)), such as in the case of astrocytic glutamate-binding presynaptic kainate receptors or group II/III metabotropic receptors (mGluRs) [13, 14, 71], or release-increasing (Figure 3(e)), when astrocytic glutamate binds NMDARs or group I mGluRs [7–9, 11, 12, 26, 72]. The functional implications of these modulations of synaptic release by glutamatergic gliotransmission on synaptic transmission have been widely addressed in a series of previous studies [18, 29, 73], and the remainder of this section reviews and extends the main results from those studies about short-term synaptic plastic and synaptic filtering.

Figure 3(d) (*left panel*) shows how postsynaptic currents (PSCs) change in the presence of release-decreasing glutamatergic gliotransmission when elicited by two consecutive action potentials arriving to the resting synapse 20 ms after the onset of gliotransmission at *t* = 5 s (Figure 3(c)). Two differences with respect to the case without gliotransmission (*black trace*) may be observed. First the PSC amplitude overall decreases (*red trace*), consistent with a decrease of synaptic efficacy caused by the reduction of synaptic release by astrocytic glutamate. Then, the second PSC is larger than the first one, which is the opposite of what would be measured in the absence of gliotransmission. In other words, in agreement with experimental observations [13], the release-decreasing effect of astrocytic glutamate results in an increased pair pulse ratio (PPR) with respect to the case without gliotransmission (PPR_0_). Notably, as shown in Figure 3(d) (*right panel*), this change in the PPR ratio is only transient and vanishes together with the effect of gliotransmission on synaptic release. Similar considerations also hold in the case of a release-increasing effect of astrocytic glutamate on synaptic transmission [8]: while PSC amplitude increases (Figure 3(f),* left panel*,* green trace*), this occurs to the detriment of PPR, which decreases instead (Figure 3(f),* right panel*). Thus, synapses whose release probability is increased by glutamatergic gliotransmission are likely to run out of neurotransmitter faster, exhibiting rapid onset of short-term depression, consistent with lower PPR values. On the contrary, synapses whose release probability is reduced by astrocyte-released glutamate deplete their neurotransmitter resources slower and may exhibit progressive facilitation (i.e., potentiation) of their efficacy to transmit action potentials and so larger PPR values [74]. That is, the plasticity mode of a synapse, namely, whether it is depressing or facilitating, may not be fixed but rather be modulated by glutamatergic gliotransmission by surrounding astrocytes in an activity-dependent fashion [29, 73].

**Figure 4:**
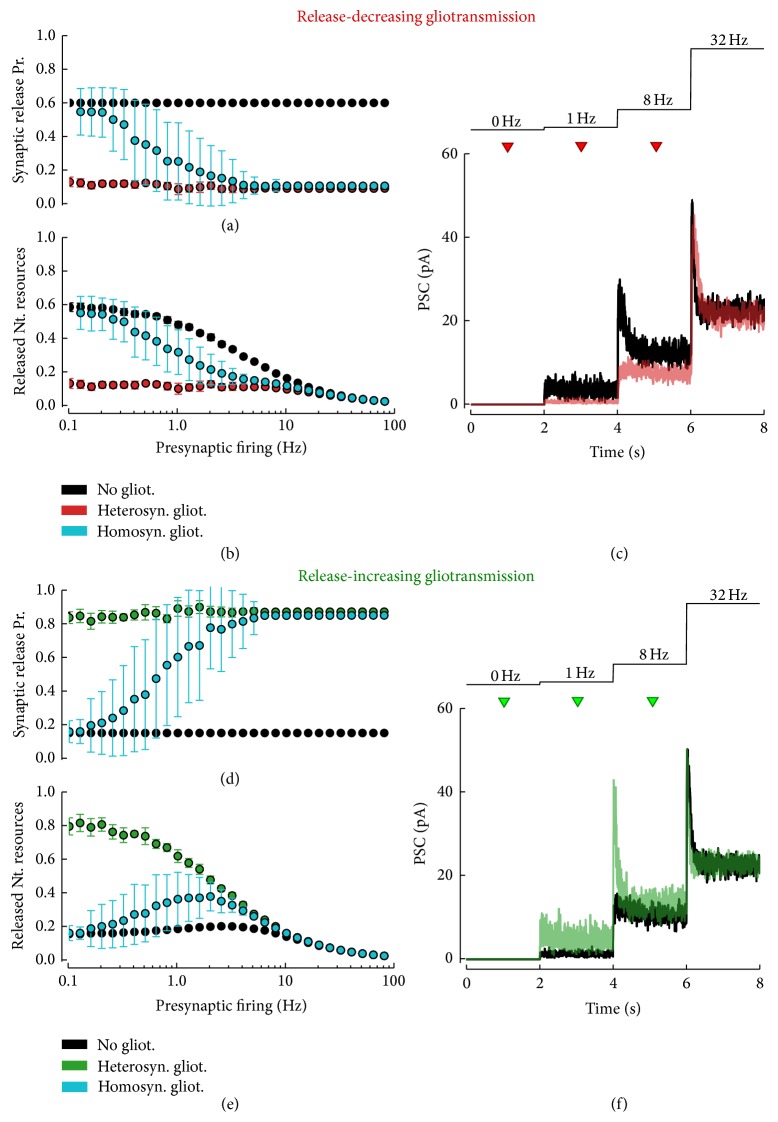
Gliotransmitter-mediated modulation of synaptic frequency response. Decrease (a) or increase (d) of synaptic release probability by gliotransmission modulates the average per-spike synaptic release, resulting in a change of the synapse frequency response. Monotonically decreasing frequency responses that are typical of depressing synapses could be flattened by release-decreasing gliotransmission ((b),* black* versus* red points*), and vice versa, almost nonmonotonic ones, characteristic of facilitating synapses, could turn into monotonically decreasing responses by release-increasing gliotransmission ((e),* black* versus* green points*). Changes in frequency response depend on whether gliotransmission impinges on the very synapse that is triggered by (homosynaptic/closed-loop scenario) or not (heterosynaptic/open-loop scenario). In the homosynaptic scenario, the synaptic response is expected to change only for presynaptic firing rates that are sufficiently high to trigger gliotransmitter release from the astrocyte ((b), (e),* cyan points*). Data points and error bars: mean ± STD for *n* = 20 (no gliot. and heterosyn. gliot.) or *n* = 200 simulations (homosyn. gliot.) with 60 s long Poisson-distributed presynaptic spike trains. ((c), (f)) The change of synaptic frequency response mediated by gliotransmission (three consecutive gliotransmitter releases at the time instants marked by* triangles*) leads to changes in how presynaptic firing rates (*top panels*) are transmitted by the synapse (*bottom panels*). Simulated postsynaptic currents (PSCs) are shown as average traces of *n* = 1000 simulations for gliotransmitter release at 1 Hz. Release-decreasing gliotransmission was achieved for *ξ* = 0, whereas *ξ* = 1 was used for release-increasing gliotransmission. Depressing synapse in ((a), (b)): *τ*_*d*_ = 0.5 s, *τ*_*f*_ = 0.3 s, and *U*_0_ = 0.6; facilitating synapse in ((d), (e)): *τ*_*d*_ = 0.5 s, *τ*_*f*_ = 0.5 s, and *U*_0_ = 0.15. Other model parameters as in Figure 3 except for *R*_in_ = 300 M*Ω*.

An important consequence of short-term synaptic dynamics is that synapses can act as filters [16, 17, 75]. Hence, modulations of synaptic dynamics by glutamatergic gliotransmission are also expected to affect the synapse's filtering characteristics [18]. This scenario is illustrated in Figure 4 where the effect of release-decreasing versus release-increasing glutamatergic gliotransmission, respectively, on depressing and facilitating synapses, is shown in terms of changes of the filtering characteristics of these synapses, that is, their steady-state release as a function of the frequency of presynaptic stimulation [17]. In the absence of gliotransmission, depressing synapses, which are characterized by intermediate-to-high initial probability of release [74] (Figure 4(a),* black circles*), predominantly act as low-pass filters (Figure 4(b),* black circles*) that are most effective at transmitting low frequency presynaptic spike trains (Figure 4(c),* black traces*). On the contrary, facilitating synapses, with a low-to-intermediate initial probability of neurotransmitter release [74] (Figure 4(a),* black circles*), function as high-pass or band-pass filters (Figure 4(b),* black circles*); that is, they are mostly effective at transmitting action potentials in an intermediate range of presynaptic activity (Figure 4(c),* black trace*).

In the presence of glutamate release by the astrocyte, these two scenarios could be reversed. Consider indeed the simple heterosynaptic case where glutamatergic gliotransmission is stimulated by other means compared to by the very synapses it impinges on. It may be noted that release-decreasing gliotransmission flattens the synaptic steady-state release towards zero for all frequencies of stimulation (Figure 4(b),* red circles*), ensuing in synaptic transmission that resembles the one of a facilitating, band-pass synapse (compare the* red* PSC* trace* in Figure 4(c) with the* black* PSC* trace* in Figure 4(f)). Vice versa, release-increasing gliotransmission could turn band-pass features of transmission by a facilitating synapse (Figure 4(e),* green circles*) into low-pass, reminiscent of a more depressing synapse (compare the* green* PSC* trace* in Figure 4(f) with the* black* PSC* trace* in Figure 4(c)). On the other hand, when gliotransmission is stimulated by the same synapses that it modulates, that is, in the homosynaptic scenario of gliotransmission, inspection of the ensuing synaptic filtering characteristics (Figures 4(b) and 4(e),* cyan circles*) reveals that these latter coincide with those obtained in the absence of gliotransmission for low frequencies of presynaptic activity, while they tend to equal those observed with heterosynaptic gliotransmission as the frequency of stimulation increases. This coexistence of mixed features from apparently opposite scenarios, that is, no gliotransmission versus heterosynaptic gliotransmission, can be explained by the fact that the release of glutamate from the astrocyte requires intracellular Ca^2+^ to cross a threshold concentration. Hence, in the homosynaptic scenario, synapses that impinge on the astrocyte must be stimulated at rate sufficiently high to allow astrocytic Ca^2+^ to increase beyond such a threshold.

The modulation of synaptic filtering by glutamatergic gliotransmission offers the possibility that the same stimulus could be differently filtered (i.e., processed) and transmitted by a synapse in the presence (or not) of glutamate release by surrounding astrocytic processes, ultimately endowing that synapse with processing versatility with respect to incoming action potentials. Moreover, to the extent that synaptic dynamics critically shape the computations performed by the neural circuitry, such versatility could also be reflected at the network level, leading to the possibility that the same neuron-glia network could be involved in different computational tasks defined, time by time, by activity-dependent gliotransmitter release by astrocytes in the network.

### 2.3. Astrocyte-Mediated Slow Inward Currents

Induction of slow inward (i.e., depolarizing) currents (SICs) by activation of extrasynaptically located postsynaptic NMDA receptors is the other mechanism considered in this study whereby glutamatergic gliotransmission could affect synaptic information transfer. While astrocyte-mediated SICs have been reported in several brain regions, the pathway underlying glutamate release by astrocytes has not been fully elucidated [76, 77]. It is likely that, similar to the presynaptic route for glutamatergic gliotransmission discussed above, multiple pathways for glutamate release could be used by the same astrocyte [39], but their deployment depends on developmental, regional, and physiological factors [27]. Astrocytic Ca^2+^ activity seems to be a crucial factor in the regulation of astrocyte-mediated SICs [19–23, 25, 78]. In particular, SIC frequency and amplitude have been shown to increase upon Ca^2+^ elevations mediated by GPCRs on astrocytes such as mGluRs [19–23, 72, 79], the metabotropic purinergic P2Y1 receptor [25], the endocannabinoid CB1 receptor [80], or the protease-activated receptor 1 (PAR1) [24]. Remarkably, stimulation of PAR1s on hippocampal astrocytes was shown to trigger, under physiological conditions, Ca^2+^-dependent glutamate release from these cells through Bestrophin-1 anion channel [81, 82], and this pathway of glutamate release has been suggested as a candidate mechanism for SICs [83]. Channel-mediated glutamate release is expected to account for prolonged (>10 s) release of transmitter but in small amounts per unit time [82] thus ensuing in modest, very slow rising and decaying inward currents. While similar SICs have indeed been recorded [71, 84], most experiments reported SICs within a wide range of amplitudes to last only few seconds at most and rise in correlation with astrocytic Ca^2+^ increases, with rise time much shorter than their decay [20–22, 24, 26, 32, 85, 86] akin to currents that would ensue from a quantal mechanism of gliotransmitter release [62].

**Figure 5:**
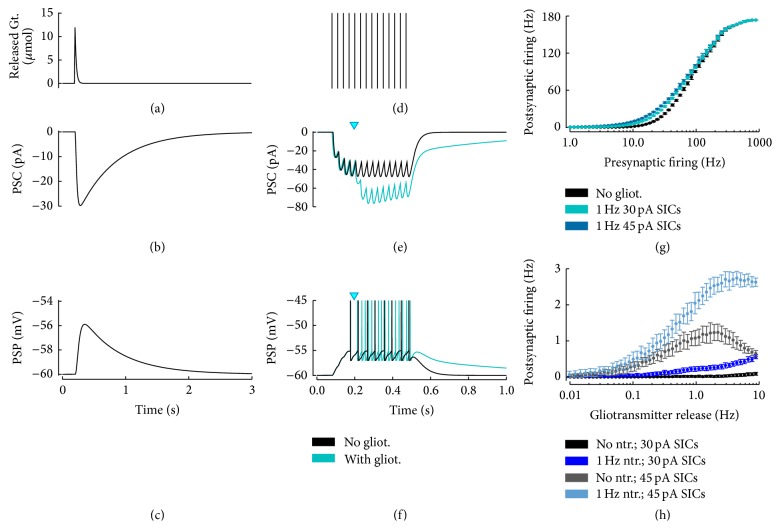
Postsynaptic pathway of gliotransmission by slow inward currents. The transient increase of gliotransmitter concentration in the perisynaptic space (a) triggers a slow inward (depolarizing) current (SIC) in the postsynaptic neuron ((b), (c)). Such SIC adds to postsynaptic currents triggered by presynaptic spikes ((d), (e),* cyan triangle* marks gliotransmitter release/SIC onset) and may dramatically alter postsynaptic firing (f). In general postsynaptic firing frequency increases with both SIC amplitude (g) and frequency (h). In this latter case, however, SICs as ample as 30 pA (similar to what reported in several experiments) need to impinge on the postsynaptic neuron at unrealistically high rates (≫0.1 Hz) in order to trigger a sensible change in the neuron's firing rate (*black data points*). Lower, more realistic SIC rates may affect neuronal firing only for larger SIC amplitudes (e.g., 45 pA,* grey data points*). The entity of SIC-mediated increase of postsynaptic neuronal firing further depends on the neuron's state of depolarization at SIC timings which is set by synaptic inputs (*blue* and* cyan data points*). Data points and error bars: mean ± STD out of *n* = 50 simulations with presynaptic Poisson-distributed spike trains. Parameters as in Table 1 except for *ϱ*_*e*_ = 10^−4^, *τ*_*e*_ = 200 ms, *τ*_*S*_^*r*^ = 10 ms, and *R*_in_ = 150 M*Ω*.

Based on these arguments, we assume glutamate exocytosis as the candidate mechanism for glutamate release by astrocytes that mediates SICs. Accordingly, we adopt the description of astrocytic glutamate exocytosis previously introduced (Figures 2(g)–2(i)) to also model astrocyte-mediated SICs. In this fashion, glutamate exocytosis by the astrocyte into the extracellular space (Figure 5(a)) results in activation of extrasynaptically located NMDARs on nearby neuronal dendrites which trigger SICs (Figure 5(b)) that cause slow depolarizing postsynaptic potentials (PSP, Figure 5(c)).

An important functional consequence of SIC-mediated depolarizations is that they can modulate neuronal excitability [21–23, 85]. As illustrated in Figures 5(d) and 5(e), astrocyte-mediated SICs (*cyan trace*) may add to regular synaptic currents (*black trace*) resulting in depolarizations of postsynaptic neurons closer to their firing threshold [23]. In turn, these larger depolarizations could dramatically change generation and timing of action potentials by those neurons in response to incoming synaptic stimuli (Figure 5(f)). This could ultimately affect several neurons within the reach of glutamate released by an astrocyte, leading to synchronous transient increases of their firing activity [21]. Remarkably, this concerted increase of neuronal excitability has often been observed in correspondence with large amplitude (i.e., >100 pA) SICs [21, 25, 85, 87], but experiments report the majority of SICs to be generally smaller, with amplitudes < 80 pA [11, 21, 22, 26, 32, 87]. It is therefore unclear whether SIC-mediated increase of neuronal excitability could occur [88] or not [87, 89, 90] in physiological conditions.

In Figure 5(g), we consider postsynaptic firing in a standard leaky integrate-and-fire neuron model [91, 92] as a function of presynaptic activity for SICs of different amplitudes (30–45 pA, see Appendix B) randomly occurring at an average rate of 1 Hz based on a binomial process for glutamate release from astrocytes as suggested by experiments [65] (see Appendix A). In line with experimental evidence [93], the input-output transfer function in the absence of gliotransmission has a typical sigmoidal shape (*black dots*) which reflects the following: (i) gradual emergence of firing for low (>10 Hz) fluctuating synaptic inputs; (ii) the progressive, quasi-linear increase of the firing rate for presynaptic activity beyond ~30 Hz; and finally, (iii) saturation of the firing rate for sufficiently strong synaptic inputs such that timing of action potential generation approaches the neuron's refractory period (which was fixed at 2 ms in the simulations, Appendix B) [92]. The addition of astrocyte-mediated SICs alters the firing characteristics of the neuron due to the ensuing larger depolarization. In particular the neuron could generate action potentials for lower rates of presynaptic activity (*cyan*/*blue dots*). Clearly, the larger the SIC is, the more the postsynaptic firing increases with respect to the case without SICs, for a given level of presynaptic activity.

As previously mentioned, these results assume an average 1 Hz rate for astrocyte-mediated SICs. While such a rate cannot be excluded, it seems unlikely for the following reasons. The weak correlation of SIC amplitude with somatic Ca^2+^ elevations observed in experiments favors indeed the idea that glutamate-mediated SICs are highly localized events, occurring within subcellular domains at astrocytic processes [22]. In turn, Ca^2+^ elevations in astrocytic processes could be as short-lived as ~0.5 s [10, 50], thus in principle allowing for glutamate release rates of the order of 1 Hz. However, in practice, reported SIC frequency is much lower, that is, <5/min (i.e., ~0.08 Hz) [11, 22]. Hence, it may be expected that the effect of SICs on neuronal firing could be considerably reduced with respect to the case in Figure 5(g).

We consider this possibility more closely in Figure 5(h), where we analyze postsynaptic firing in function of the average frequency of astrocyte-mediated SICs, both in the absence of synaptic activity (*black* and* dark blue dots*) and in the case of presynaptic activity at an average rate ~ 1 Hz, which corresponds to typical levels of spontaneous activity in vivo [94] (*grey* and* light blue dots*). It may be noted that the effect of SICs of typical amplitudes on postsynaptic firing rate is generally small, that is, <0.5 Hz, except for unrealistic (>0.1 Hz) SIC rates, while it gets stronger in association with synaptic activity. In this latter case however the possible increase in postsynaptic firing by astrocyte-mediated SICs is limited by the rate of reintegration of released glutamate resources in the astrocyte (fixed at ~1 Hz, Appendix B). Analogously to short-term synaptic depression in fact, our description of gliotransmitter release predicts that, for release rates that exceed the rate of reintegration of released glutamate by the astrocyte, exhaustion of astrocytic glutamate resources available for further release will result in SICs of smaller amplitude. In this fashion, due to depletion of astrocytic glutamate, the effect of large rates of glutamate release and thus of SICs on neuronal firing tends to be equivalent to that of considerably lower ones.

Taken together, the above results do not exclude a possible role of SICs in modulation of neuronal excitability and firing but suggest that such modulation could effectively occur only in coincidence with proper levels of synaptic activity. In this fashion, astrocyte-mediated SICs could be regarded to operate a sort of coincidence detection between synaptic activity and astrocytic glutamate release [22], whose readout would then be a temporally precise, cell-specific increase of neuronal firing (Figure 5(f)).

## 3. Astrocyte-Mediated Regulation of Long-Term Plasticity

The strength of a synaptic connection between two neurons can be modified by activity, in a way that depends on the timing of neuronal firing on both sides of the synapse, through a series of processes collectively known as spike-timing-dependent plasticity (STDP) [95]. As both pre- and postsynaptic pathways of glutamatergic gliotransmission potentially change EPSC magnitude, thereby affecting postsynaptic firing, it may be expected that they could also influence STDP.

Although the molecular mechanisms of STDP remain debated, and different mechanisms could be possible depending on type of synapse, age, and induction protocol [34], at several central excitatory synapses postsynaptic calcium concentration has been pointed out as a necessary factor in induction of synaptic changes by STDP [31, 96–99]. Remarkably, amplitude and, likely, time course of postsynaptic Ca^2+^ could control the direction of plasticity: smaller, slower increases of postsynaptic Ca^2+^ give rise to spike-timing-dependent long-term depression (LTD), whereas larger, more rapid increases cause spike-timing-dependent long-term potentiation (LTP) [31, 96, 97]. In calcium-based STDP models, this is also known as the “Ca^2+^-control hypothesis” [35, 100, 101]. According to this hypothesis, no modification of synaptic strength occurs when Ca^2+^ is below a threshold *θ*_*d*_ that is larger than the resting Ca^2+^ concentration. If calcium resides in an intermediate concentration range, between *θ*_*d*_ and a second threshold *θ*_*p*_ > *θ*_*d*_, the synaptic strength is decreased. Finally, if calcium increases above the second threshold, *θ*_*p*_, the synaptic strength is potentiated.

**Figure 6:**
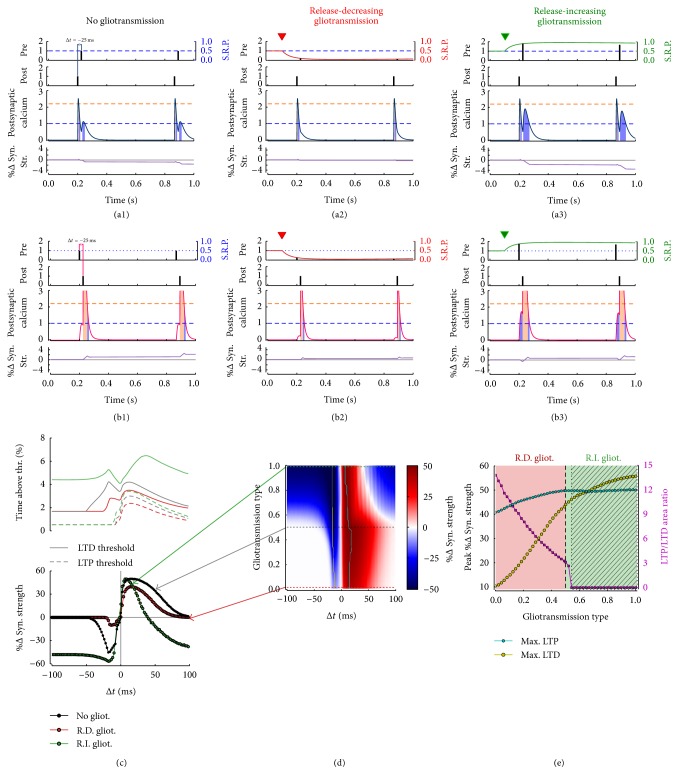
STDP modulation by gliotransmitter regulation of synaptic release. ((a), (b)) Rationale of LTD and LTP without ((a1), (b1)) and with either release-decreasing ((a2), (b2), *ξ* = 0) or release-increasing gliotransmission ((a3), (b3), *ξ* = 1) setting on at the* red*/*green marks*. (c) Percentage of time spent by postsynaptic Ca^2+^ transients (*left panel*) above depression (*dashed lines*) and potentiation thresholds (*solid lines*) for spike-timing intervals (Δ*t*) within ±100 ms and resulting STDP curves (*right panel*) in the absence of gliotransmission (no gliot.,* black curve*) and with maximal release-decreasing (RD,* red curve*) or release-increasing gliotransmission (RI,* green circles*). (d) In general, strength and direction (i.e., “type”) of gliotransmission may dramatically modulate STDP. For example, synaptic changes are attenuated when synaptic release is decreased by gliotransmission (area below the* black dashed line*). Conversely, for sufficiently strong release-increasing gliotransmission (area above the* black dotted line*), the LTP window shrinks and LTD may be measured for all Δ*t* < 0, as well as for sufficiently large Δ*t* > 0. (e) A closer inspection of STDP curves indeed reveals that LTD (*yellow curve*) increases for larger synaptic release accounted by gliotransmission, while the ratio between areas underneath the LTP and LTD (*magenta curve*), initially in favor of the former (i.e., for release-decreasing gliotransmission), reduces to zero for large enough release-increasing gliotransmission, when two open LTD windows appear outside a small LTP window center for small Δ*t* > 0 (*hatched area*). Synaptic parameters: *τ*_*d*_ = 0.33 s, *τ*_*f*_ = 0.33 s, and *U* = 0.5 s. Other parameters as in Table 1 except for *ϱ*_*e*_ = 10^−4^, *τ*_*c*_ = 1 ms, *W*_*N*_ = 78.7, *τ*_*P*_ = 5 s in ((a), (b)), and *τ*_*P*_ = 30 s otherwise.

Figures 6(a1) and 6(b1) exemplify the operational mechanism of the Ca^2+^-control hypothesis within the framework of a nonlinear Ca^2+^-based model for STDP at glutamatergic synapses originally introduced by Graupner and Brunel [102]. At most glutamatergic synapses, postsynaptic Ca^2+^ is mainly regulated by two processes: (i) postsynaptic Ca^2+^ entry mediated by NMDARs [103] and (ii) Ca^2+^ influx by voltage-dependent Ca^2+^ channels (VDCCs) [31, 33, 99, 104]. In this fashion, each presynaptic action potential generates a long-lasting Ca^2+^ transient by opening NMDAR channels, while postsynaptic firing results in a short-lasting Ca^2+^ transient due to opening of VDCCs by dendritic depolarization through back-propagating action potentials (bAPs) [95]. Presynaptic action potentials alone do not trigger changes in synaptic strength, but they do so in correlation with postsynaptic bAPs [105]. Notably [106], in a typical STDP induction pairing protocol, LTD is induced if the postsynaptic neuron fires before the presynaptic one, that is, post → pre pairing at negative spike-timing intervals Δ*t* (Figure 6(a1)). Contrarily, LTP is induced when the presynaptic cell fires before the postsynaptic cell, that is, for pre → post pairing at positive Δ*t* intervals (Figure 6(a1)). This is possible because, when a presynaptic action potential is followed shortly after by a postsynaptic bAP, the strong depolarization by this latter drastically increases the voltage-dependent NMDAR-mediated Ca^2+^ current due to removal of the NMDAR magnesium block [107, 108], thereby resulting in supralinear superposition of the NMDAR- and VDCC-mediated Ca^2+^ influxes.

In the framework of the Ca^2+^-control hypothesis, these observations may be summarized as follows. For large Δ*t*, pre- and postsynaptic Ca^2+^ transients do not interact, and the contributions from potentiation and depression by pre/post pairs (or vice versa) cancel each other, leading to no synaptic changes on average (Figure 6(c),* black curves*). For short, negative Δ*t*, the presynaptically evoked Ca^2+^ transient rises instead above the depression threshold (*θ*_*d*_) but not beyond the potentiation threshold (*θ*_*p*_). Consequently, depression increases whereas potentiation remains constant, which leads to LTD induction. For short, positive Δ*t* however the postsynaptically evoked calcium transient rises on top of the presynaptic transient by the NMDAR nonlinearity and increases activation of both depression and potentiation. Because the rate of potentiation is larger than the rate of depression (Appendix C), this results in LTP induction.

For the same number of pre/post pairs (or vice versa), mapping of the average synaptic modification as a function of the spike-timing interval Δ*t* ultimately provides an STDP curve that qualitatively resembles the classic curve originally described by Bi and Poo [123] (Figure 6(c),* right panel*,* black curve*). In the following, we will focus on parameters that lead to such a STDP curve and investigate how this curve is affected in the presence of glutamatergic gliotransmission, through the pre- and postsynaptic pathways of regulation discussed above.

### 3.1. Presynaptic Pathway

The very nature of synaptic transmission crucially depends on the synapse's initial probability of neurotransmitter release, insofar as this latter sets both the tone of synaptic transmission, that is, how much neurotransmitter is released per action potential by the synapse on average, and whether the synapse displays short-term depression or facilitation [17]. Synapses with low-to-intermediate values of initial neurotransmitter release probability, for example, Schaffer collateral synapses [74], or some cortical synapses [16], are indeed prone to display facilitation, whereas synapses that are characterized by large release probability are generally depressing [16]. Because synaptic release probability also dictates the degree of activation of NMDARs and consequently the magnitude of postsynaptic Ca^2+^ influx, it is expected that both the tone of synaptic transmission and its short-term dynamics could affect STDP [34]. The relative weight of these two factors in shaping synaptic changes however likely depends on the protocol for STDP induction. Short-term plasticity could indeed sensibly regulate STDP induction only for rates of presynaptic action potentials high enough to allow facilitation or depression of synaptic release from one AP to the following one [109, 110]. In this study, we consider low pre/post frequencies of 1 Hz. At such frequencies we expect short-term plasticity to have a negligible effect, and thus we only focus on how changes in the tone of synaptic transmission by glutamatergic gliotransmission affect STDP.

Figures 6(a2) and 6(b2), respectively, show the outcome of LTD and LTP induction for two consecutive pre → post and pre → post pairings preceded by the onset of release-decreasing gliotransmission at 0.1 s (*top panels*,* black marks*). A comparison of the ensuing postsynaptic Ca^2+^ dynamics with respect to the case without gliotransmission (Figures 6(a1) and 6(b1)) reveals that the strong decrease of synaptic release probability (SRP,* top panels*,* red curves*) caused by gliotransmission remarkably reduces the NMDAR-mediated contribution to postsynaptic Ca^2+^ influx (*middle panels*), resulting in smaller variations of synaptic strength (*bottom panels*). In this fashion, at the end of the pairing protocol, release-decreasing gliotransmission accounts for less time spent by Ca^2+^ above both LTD and LTP thresholds (Figure 6(c),* left panel*,* red traces*). The resulting STDP curve thus displays strongly attenuated LTD and LTP (Figure 6(c),* right panel*,* red curve*), with LTP windows spanning a considerably smaller range of Δ*t*s values than in the curve obtained without gliotransmission (*black curve*).

Similar considerations apply to the case of release-increasing gliotransmission (Figures 6(a3) and 6(b3)). In this case, the NMDAR component of postsynaptic Ca^2+^ could increase by gliotransmission even beyond the *θ*_*d*_ threshold (*dashed blue line*), thus favoring depression while reducing potentiation (*bottom panels*). In particular, for short positive Δ*t*, the maximal LTP does not change but the Δ*t* range for LTP induction shrinks. For Δ*t* > 40 ms in fact, the time that Ca^2+^ spends above the LTD threshold increases with respect to the time spent by Ca^2+^ above the LTP threshold, thereby resulting in LTD induction (Figure 6(c),* left panel*,* green traces*). In this fashion, the STDP curve in the presence of release-increasing gliotransmission displays a narrow 0–40 ms LTP window outside which LTD occurs instead (Figure 6(c),* right panel*,* green curve*).

Figure 6(d) summarizes how the STDP curve changes for the whole spectrum of glutamatergic gliotransmission. In this figure, a *y*-axis value of “gliotransmission type” equal to 0 corresponds to maximum release-decreasing gliotransmission (*red curve* in Figure 3(c)); a value equal to 1 stands instead for maximum release-increasing gliotransmission (as in the case of the* green curve* in Figure 3(c)); finally, a value of 0.5 corresponds to no effect of gliotransmission on synaptic release (*black curve* in Figure 3(c)). It may be noted that gliotransmission may affect the STDP curve in several ways, changing both strength of plastic changes (*color code*) and shape and areas of LTP and LTD windows. In particular, as revealed by Figure 6(e), maxima of LTP (*cyan circles*) and LTD (*yellow circles*) decrease with decreasing values of gliotransmission type, consistently with smaller postsynaptic Ca^2+^ influx for larger decreases of synaptic release by gliotransmission. This suggests that release-decreasing gliotransmission (*red-shaded area*) could attenuate STDP yet in a peculiar fashion, counteracting LTD more than LTP induction, as reflected by increasing values of LTP/LTD area ratio (*magenta curve*).

On the contrary, the effect of release-increasing gliotransmission (Figure 6(e),* green-shaded area*) could be dramatically different. For sufficiently strong increases of synaptic release by gliotransmission in fact, the LTP/LTD area ratio drops to zero (*hatched area*) in correspondence with the appearance of two “open” LTD windows, one for Δ*t* < 0 and the other for sufficiently large positive spike-timing intervals. In parallel, consistently with the fact that release-increasing gliotransmission tends to increase the fraction of time spent by postsynaptic Ca^2+^ above the threshold for LTD thereby promoting this latter (Figure 6(c)), the range for LTP induction also tends to shrink to lower Δ*t* values as release-increasing gliotransmission grows stronger (Figure 6(d),* red color-coded areas* for gliotransmission type > 0.5).

In summary, our analysis reveals that modulation of synaptic release by glutamatergic gliotransmission could change STDP both quantitatively and qualitatively, from hindering its induction for release-decreasing modulations to altering both shape and existence of LTD windows for release-increasing modulations. However, whether and how this could effectively be observed in experiments remain to be investigated. Supported both by experimental evidence and theoretical arguments is the notion that regulations of the tone of synaptic transmission by glutamatergic gliotransmission likely require specific morphological and functional constraints to be fulfilled by the nature of astrocyte-synapse coupling [5, 18]. Similar arguments may ultimately hold true also for modulation of STDP; insofar as for this modulation to be measured in our simulations, we required both a sufficiently strong increase/decrease of synaptic release by gliotransmission and a decay time of such increase/decrease long enough for this latter to be present during the induction protocol. Should these two aspects not have been fulfilled in our simulations, then modulation of STDP by gliotransmitter-mediated changes of synaptic release would likely have been negligible or even undetectable.

### 3.2. Postsynaptic Pathway

We now turn our analysis to the possible impact of astrocyte-mediated SICs on STDP. Because SICs are through extrasynaptic NMDA receptors and these receptors are mainly permeable to Ca^2+^ ions [111], then SICs could contribute to postsynaptic Ca^2+^ thereby affecting STDP. Nevertheless, we should note that it is unclear whether and how extrasynaptic NMDARs contribute to plasticity, independently of the occurrence of SICs [77]. For example, theta-burst LTP induction in CA1 neurons of rat hippocampal slices is turned into LTD when extracellular NMDARs are selectively stimulated [112], but it is unknown whether these receptors have a role in STDP [113]. In general, for a given STDP induction protocol, two factors that could crucially regulate how Ca^2+^ transients mediated by extrasynaptic NMDARs are involved in STDP are the location of these receptors on the spine and the morphology of this latter in terms of spine head and neck [114, 115]. Unfortunately both these factors remain unknown in the current knowledge of SIC-mediating extrasynaptic NMDARs and, for the remainder of this study, we assume that, in spite of their possible location away from the postsynaptic density along the spine neck or the dendritic shaft [116], SIC-mediating extrasynaptic NMDARs could still regulate spine Ca^2+^ dynamics [27].

**Figure 7:**
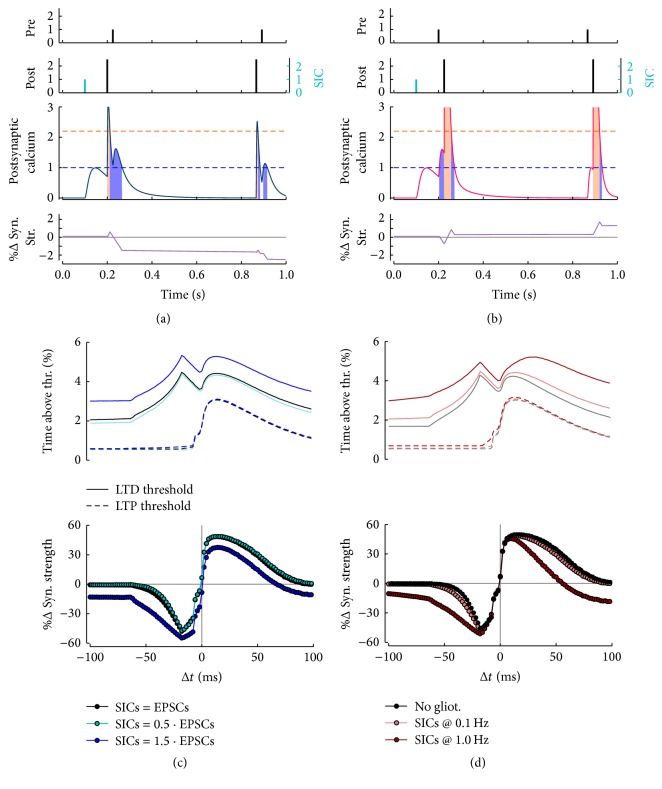
STDP modulation by gliotransmitter-mediated SICs. ((a), (b)) Inspection of postsynaptic Ca^2+^ in the initial part of a pairing protocol that includes a gliotransmitter-mediated slow inward current (SIC) arriving to the postsynaptic neuron at *t* = 0.1 s illustrates how SICs have the potential to modulate postsynaptic Ca^2+^ thereby regulating LTD and LTP. (c) The magnitude of modulation depends on how large SICs are with respect to synaptic inputs (EPSCs) as well as at (d) what rate they occur. ((c), (d)) STDP curves were calculated for 60 pre/post pairings at 1 Hz and included SICs starting 0.1 s before the first pairing and occurring at 0.1 Hz. Synaptic parameters: *τ*_*d*_ = 0.33 s, *τ*_*f*_ = 0.33 s, and *U*_0_ = 0.5 s. Other parameters as in Table 1 except for *ϱ*_*e*_ = 10^−4^, *τ*_*c*_ = 1 ms, *τ*_*e*_ = 200 ms, *τ*_*S*_^*r*^ = 5 ms, and *τ*_*S*_ = 100 ms.

Based on the above rationale, we thus model SICs as slow postsynaptic Ca^2+^ transients that will add to presynaptically and postsynaptically triggered ones and study their effect on the induction of SDTP by classic pairing protocols. For the sake of generality, we express the peak of SIC-mediated Ca^2+^ transients in units of NMDAR-mediated EPSCs. However, since in our STDP description individual EPSCs do not trigger any synaptic modification [102], then we may expect that only SICs sufficiently larger than EPSCs could effectively affect STDP. On the other hand, smaller SICs could also combine with Ca^2+^ transients by pre/post pairings, resulting in Ca^2+^ elevations beyond either LTD or LTP thresholds that would ultimately cause synaptic changes (Figures 7(a) and 7(b)). Hence, based on these considerations, we deem amplitude and timing of SICs, in terms of both frequency of occurrence and onset with respect to STDP-inducing stimuli, to be crucial factors in shaping how SICs affect STDP, and thus we set to analyzing these three factors separately.

Figure 7(c) summarizes the results of our simulations for SICs as large as 0.5, 1, or 1.5 times typical EPSCs, occurring at a fixed rate of 0.1 Hz and starting 100 ms before the delivery of 60 STDP-inducing pre/post pairings at 1 Hz. As illustrated in Figures 7(a) and 7(b), for the same SIC kinetics, these simulations guarantee superposition between Ca^2+^ influxes mediated by SICs and pre/post pairings such that the extension of the ensuing Ca^2+^ transient beyond LTD and LTP thresholds (*dashed lines*) merely depends on SIC amplitude. In this fashion, it may be noted that SICs of amplitude smaller than or equal to typical EPSCs (Figure 7(c),* turquoise circles* and* black circles,* resp.), which alone would not produce any synaptic modification, do not sensibly change the STDP curve with respect to the previously considered case of an alike synapse in the absence of gliotransmission (Figure 6(c),* black circles*). Conversely, large SICs could dramatically affect STDP, shifting the STDP curve towards negative synaptic changes (*blue circles*), and this negative shift increases the larger SICs grow beyond the *θ*_*d*_ threshold (results not shown). In this case, STDP generally results in LTD with the exception of a LTP window that is comprised between ~0 ms and positive Δ*t* values that are smaller than those in the absence of gliotransmission (Figure 6(c),* green circles*). This resembles what was previously observed for STDP curves in the presence of release-increasing gliotransmission, with the only difference that, for large |Δ*t*| values, LTD strength in the presence of astrocyte-mediated SICs is found to be the same, regardless of Δ*t* (compare the* blue curve* in Figure 7(c) with the* green curve* in Figure 6(c)).

In Figure 7(d) we consider the alternative scenario where only SICs as large as typical EPSCs impinge on the postsynaptic neuron at different rates, yet always 100 ms before STDP-inducing pairings. Akin to what happens for SIC amplitudes, the larger the SIC frequency is, the more the STDP curve changes. Indeed, as SIC frequency increases above SIC decay rate (i.e., 1/*τ*_*A*_, Appendix A, Section A.1.4), SIC-mediated Ca^2+^ transients start adding up, so that the fraction of time spent by Ca^2+^ beyond the LTD threshold increases favoring LTD induction. In this fashion, the ensuing STDP curve, once again, consists of a narrow LTP window for Δ*t* ≥ 0, outside which only LTD is observed (*red curve*). In practice however, because SICs occur at rates that are much slower than their typical decay (Appendix B), they likely affect STDP in a more subtle fashion. This may be readily understood considering the* pink* STDP* curve* obtained for SICs at 0.1 Hz, that is, the maximum rate experimentally recorded for these currents [22]. Inspection of this curve indeed suggests that SICs could effectively modulate LTD and LTP maxima as well as the outer sides of the LTD/LTP windows, which dictate how fast depression/potentiation decay for large |Δ*t*|, but overall the qualitative features of the STDP curve are preserved with respect to the case without gliotransmission (*black curve*).

**Figure 8:**
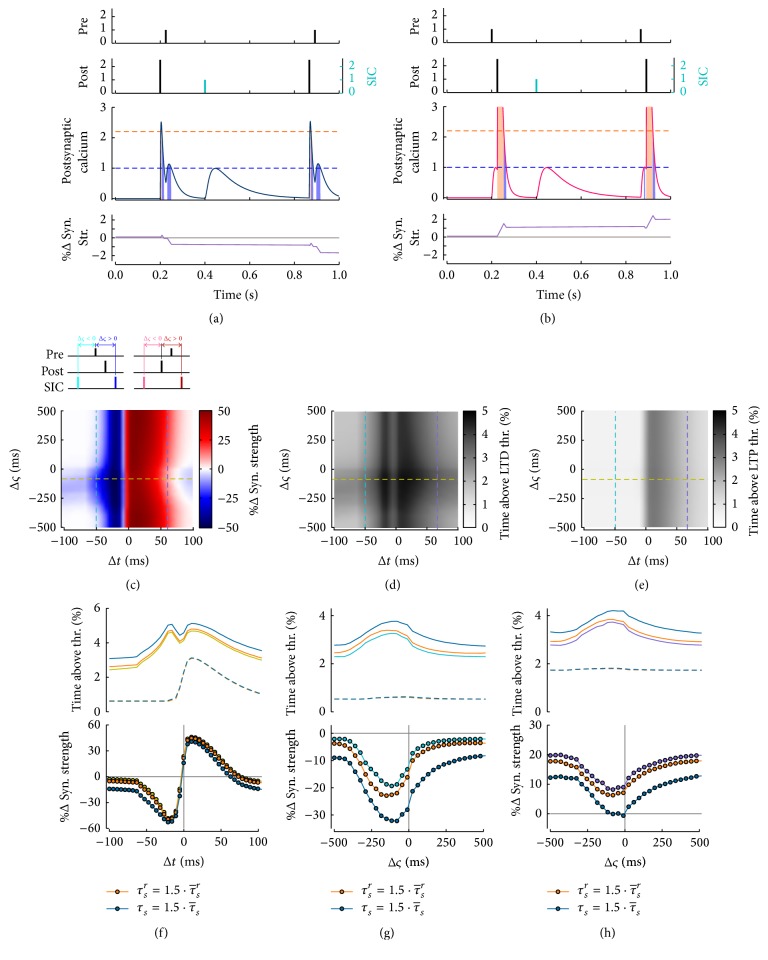
Effect on STDP of SIC timing with respect to pairings. ((a), (b)) Impact on plasticity of a SIC occurring 0.2 ms after the first pairing instead of 0.1 s before it as previously considered in Figures 7(a), 7(b). (c) STDP curves as a function of the SIC pre/post pair delay (Δ*ς*) show how LTD could get stronger while the LTP window shrink for small-to-intermediate Δ*ς* ≤ 0 in correspondence with ((d), (e)) a maximum of the duration of Ca^2+^ transients above the LTD threshold. These results were obtained assuming SIC rise and decay time constants, respectively, equal to $\tau_{s}^{r}={\bar{\tau }}_{s}^{r}=20\;$ms and $\tau_{s}={\bar{\tau }}_{s}=200\;$ms. ((f), (h)) Peak and range of this LTD increase ultimately depend on SIC kinetics as reflected by the change of sample curves for specific Δ*ς* (*yellow curve*) and spike-timing intervals (*cyan* and* purple curves*) when SIC rise and/or decay time constants were slowed down 1.5-fold (*orange* and* blue curves,* resp.). ((c), (h)) The same pairing protocol of Figures 7(c) and 7(d) was used but with a SIC frequency of 0.2 Hz and variable SIC onset and kinetics according to Δ*ς*, *τ*_*S*_^*r*^, and *τ*_*S*_. Parameters as in Figure 7.

Clearly, the extent of the impact of SIC amplitude and frequency on STDP discussed in Figures 7(c) and 7(d) ultimately depends on when SICs occur with respect to ongoing STDP-inducing pairings. Had we set SICs to occur ~200 ms after pre/post Ca^2+^ transients in our simulations, then, as illustrated in Figures 8(a) and 8(b), we would have not detected any sensible alteration of STDP, unless SICs were larger than typical EPSCs and/or occurred at sufficiently high rate to generate Ca^2+^ transients beyond the plasticity thresholds (results not shown). To seek understanding of how timing of SICs versus pre/post pairings could alter LTD and/or LTP, we simulated STDP induction by pairing as the time interval (Δ*ς*) between SIC and pre/post pairs was systematically varied (with SIC rate fixed at 0.2 Hz) (Figures 8(c)–8(e)). In doing so, we adopted the convention that negative Δ*ς* values stand for SICs preceding pre/post (or post/pre) pairings while positive Δ*ς* values refer to the opposite scenario of SICs that follow pairings (Figure 8(c),* top schematics*). Then, it may be observed that, for Δ*ς* approximately in between −300 ms and 0 ms, LTD could be induced for any negative Δ*t* as well as for large positive Δ*t* (Figure 8(c),* blue tones*), in this latter case to the detriment of the LTP window, whose upper bound moves to lower Δ*t* values (Figure 8(c),* red tones*). This results in STDP curves (e.g., Figure 8(f),* yellow curve* for Δ*ς* = −75 ms) that bear strong analogy with the blue and red curves in Figures 7(c) and 7(d), respectively, obtained for SICs of large amplitude and frequency and suggest that depression grows as SICs tend to concur with pre/post pairings. An inspection of postsynaptic Ca^2+^ transients (Figures 8(d) and 8(e)) indeed reveals that coincidence of SICs and pre/post pairings, which occurs at negative Δ*ς* of the order of SIC rise time (see Appendix B), corresponds to the longest time spent by Ca^2+^ above the LTD threshold, thereby resulting in maximum LTD (Figure 8(g)) and thus minimum LTP (Figure 8(h)). Clearly, the Δ*ς* range for which coincidence of SICs with pre/post pairings enhances LTD induction ultimately depends on kinetics of SICs, as reflected by their rise (*τ*_*s*_^*r*^) and/or decay time constants (*τ*_*s*_), and spans Δ*ς* values approximately comprised within ± SIC duration (i.e., ≃*τ*_*s*_^*r*^ + *τ*_*s*_). As SIC duration increases in fact, because of either larger *τ*_*s*_^*r*^ or larger *τ*_*s*_ or both, so does the Δ*ς* range for LTD enhancement, as reflected by the* orange* and* blue curves* in Figures 8(f)–8(h).

In conclusion the simulations in Figures 8(c)–8(h) point to both timing and duration of SICs with respect to pre/post pairing-mediated Ca^2+^ transients as a further, potentially crucial factor in setting strength and polarity of STDP at glutamatergic synapses. It is noteworthy to emphasize that, however, to appreciate some effect on STDP, we had to assume in those simulations SICs occurring at 0.2 Hz, that is, twofold the maximum SIC rate (i.e., ~0.1 Hz) experimentally observed [22]. Indeed, analogous simulations run with realistic SIC rates ≤ 0.1 Hz did produce only marginal changes to STDP curves, akin to those previously observed for the* pink* STDP curve in Figure 7(d). The potential functional implications (or lack thereof) of this perhaps puzzling result are addressed in Discussion.

## 4. Discussion

A large body of evidence has accumulated over the last years suggesting an active role of astrocytes in many brain functions. Collectively, these data fuelled the concept that synapses could be tripartite rather than bipartite, since in addition to the pre- and postsynaptic terminals, the astrocyte could be an active element in synaptic transmission [1, 49, 117]. Using a computational modeling approach, we showed here that glutamatergic gliotransmission could indeed play several roles in synaptic information transfer, either modulating synaptic filtering or controlling postsynaptic neuronal firing, as well as regulating both short- and long-term forms of synaptic plasticity. Supported by experimental observations [8, 9, 13, 23, 118], these results complement and extend previous theoretical work on astrocyte-mediated regulations of synaptic transmission and plasticity [18, 29] and pinpoint biophysical conditions for a possible role of glutamatergic gliotransmission in spike-timing-dependent plasticity.

An important prediction of our model indeed is that both pathways of regulation of synaptic transmission by astrocytic glutamate considered in this study, presynaptic modulation of transmitter release and postsynaptic SICs, could affect STDP, potentially altering induction of LTP and LTD. This alteration could encompass changes in the timing between pre- and postsynaptic firing that is required for plasticity induction, as well as different variations of synaptic strength in response to the same stimulus. With this regard, the increase of LTP observed in our simulations, when moving from release-decreasing to release-increasing gliotransmission (Figure 6(e)), agrees with the experimental observation that LTP induction at hippocampal synapses requires weaker stimuli in the presence of endogenous glutamatergic gliotransmission rather than when gliotransmission is inhibited thereby decreasing synaptic release probability [9].

Notably, spike-timing-dependent plasticity in the hippocampus is not fully understood insofar as STDP induction by pairing protocols has produced a variety of seemingly contradicting observations for this brain region [119]. Recordings in hippocampal slices, for example, showed that pairing of single pre- and postsynaptic action potentials at positive spike-timing intervals could trigger LTP [120–122], as effectively expected by the classic STDP curve [123], but also induce either LTD [124] or no plasticity at all [121]. Although different experimental and physiological factors could account for these diverse observations [119, 125], we may speculate that glutamatergic gliotransmission by astrocytes, which in those experiments was not explicitly taken into account, could also provide an alternative explanation. For example, the prediction of our model that release-increasing glutamatergic gliotransmission could account for multiple LTD windows, at either positive or negative spike-timing intervals (Figure 6), indeed supports the possibility that LTD in the hippocampus could also be induced by proper presentations of pre → post pairings sequences [124]. On the same line of reasoning, the possibility that astrocyte-mediated SICs could transiently increase postsynaptic firing (Figure 5(f)) could explain why, in some experiments, precise spike timing in the induction of synaptic plasticity in the hippocampus could exist only when single EPSPs are paired with postsynaptic bursts [124, 126]. Moreover, it was also shown that postsynaptic firing is relatively less important than EPSP amplitude for the induction of STDP in the immature hippocampus compared to the mature network, possibly due to a reduced backpropagation of somatic APs in juvenile animals [121]. Remarkably, these diverse modes of plasticity induction could also ensue from different dynamics of glutamatergic gliotransmission, as likely mirrored by the developmental profile of somatic Ca^2+^ signals in hippocampal astrocytes [47], which have been reported to be much more frequent in young mice [127]. Insofar as somatic Ca^2+^ signals may result in robust astrocytic glutamate release that could trigger, in turn, similar increases of synaptic release and/or SICs [5, 62], the frequent occurrence of these latter could then ultimately guarantee a level of dendritic depolarization sufficient to produce LTP in mice pups [128].

High amplitude/rate SICs, or large increases of synaptic release mediated by glutamatergic gliotransmission, result, in our simulations, in LTD induction for any spike-timing interval except for a narrow LTP window at small-to-intermediate Δ*t* > 0. This is in stark contrast with STDP experiments, where the observed plasticity always depends, to some extent, on the coincidence of pre- and postsynaptic activity, as EPSPs or postsynaptic action potentials fail to induce plasticity by their own [95, 105]. Apart from the consideration that large SIC amplitudes/rates and large increases of synaptic release by astrocytic glutamate may not reflect physiological conditions [76, 90], this contrast may be further resolved on the basis of the following arguments.

A first consideration is that we simulated plasticity induction assuming either persistent occurrence of SICs or continuous modulations of synaptic release during the whole induction protocol. While this rationale proved useful to identify the possible mechanisms of regulation of STDP by glutamatergic gliotransmission, it may likely not reflect what occurs in reality. Indeed, modulations of synaptic release by glutamatergic gliotransmission could last only few tens of seconds [7, 8] and thus be short-lived with respect to typical induction protocols which are of the order of minutes [33, 105, 129]. Moreover, the morphology of astrocytic perisynaptic processes is not fixed but likely undergoes dynamical reshaping in an activity-dependent fashion during plasticity induction [118, 130], thereby potentially setting time and spatial range of action of gliotransmission on nearby synaptic terminals [18]. In this fashion, LTD for large spike-timing intervals could be induced only transiently and at selected synapses, focally targeted by glutamatergic gliotransmission, while leaving unchanged the qualitative features of the classic STDP curve obtained by somatic recordings in the postsynaptic neuron [129].

A further aspect that we did not take into account in our simulations is also the possible voltage dependence of astrocyte-triggered SICs. The exact nature of this dependence remains to be elucidated and likely changes with subunit composition of NMDA receptors that mediate SICs in different brain regions and at different developmental stages [77]. Regardless, it may be generally assumed that slow inward currents through NMDA receptors become substantial only for intermediate postsynaptic depolarizations when the voltage-dependent Mg^2+^ block of these receptors is released [108]. In this fashion, the possible effect of SICs on STDP would be confined in a time window around Δ*t* ≥ 0 for which coincidence with pre- and postsynaptic spikes allows for robust depolarization of postsynaptic spines. Outside this window instead, SICs would be negligible, and plasticity induction would essentially depend on mere pre- and postsynaptic spiking rescuing the experimental observation of no synaptic modification for large spike-timing intervals [95, 105].

On the other hand, even without considering voltage dependence of SIC-mediating NMDARs, the precise timing of SICs with respect to pre/post pairs is predicted by our analysis, to be potentially critical to determine strength and sign of plasticity. Similar considerations could also hold for the onset time and duration of modulations of synaptic release triggered by gliotransmission with respect to the temporal features of plasticity-inducing stimuli [29]. This ultimately points to timing of glutamate release by the astrocyte (and its downstream effects on synaptic transmission) as a potential additional factor for associative (Hebbian) learning, besides sole correlation between pre- and postsynaptic activities [131, 132]. Remarkably, this could also provide a framework to conciliate the possibility that modest, sporadic SICs that we predict would not substantially affect STDP (Figure 7) could do so instead [32]. Indeed our predictions are based on the average number of SICs within a given time window, as documented in literature, rather than on the precise timing of those SICs in that time window. In this fashion, for example, there is no distinction in terms of effect on STDP in our simulations, between a hypothetical scenario of three SICs randomly occurring on average every ~10 s in a 30 s time frame and the alternative scenario of three SICs taking place within the same time frame but in rapid succession (Figure 5b in [22]), as could happen following an exocytic burst of glutamate release by the astrocyte [61, 62, 65]. Yet the latter case could result in a dramatically different plasticity outcome with respect to the former. While individual SICs likely fail to induce synaptic modification alone in fact, their occurrence in rapid succession would instead allow postsynaptic Ca^2+^ levels to quickly increase beyond one of the thresholds for plasticity induction. Furthermore, this increase could further be boosted by coincidence of SICs with pre- and postsynaptic activity, ultimately accounting for robust LTP, as indeed predicted by other theoretical investigations [36]. However, to complicate this intriguing scenario is the observation that glutamatergic gliotransmission [65] and even more so astrocyte-mediated SICs [19, 25] are likely not deterministic but rather stochastic processes. Therefore, it would ultimately be interesting to understand how this stochasticity could affect neuronal activity and shape learning [133].

To conclude, our analysis provides theoretical arguments in support of the hypothesis that, beyond neuronal firing, astrocytic gliotransmission could represent an additional factor in the regulation of activity-dependent plasticity and learning [18, 134, 135]. This could occur in a variegated fashion by both presynaptic and postsynaptic elements targeted by glutamatergic gliotransmission, with possibly diverse functional consequences. Nonetheless, the practical observation in future experiments of a possible mechanism of action of glutamatergic gliotransmission on activity-dependent plasticity will depend on the implementation of novel specific plasticity-inducing protocols that match possible stringent temporal and spatial dynamical constraints defining the complex nature of neuron-astrocyte interactions.

## Figures and Tables

**Table 1:** 

Symbol	Description	Range	Value	Units
Synaptic dynamics				
*τ*_*d*_	Depression time constant	>0.01–2	s.s.	s
*τ*_*f*_	Facilitation time constant	>0.5–2	s.s.	s
*U*_0_	Resting synaptic release probability	<0.09–0.9	s.s.	—

Neurotransmitter release and time course				
*Y*_*T*_	Total vesicular glutamate concentration	300–1000	500	mM
*ϱ*_*c*_	Vesicular versus mixing volume ratio		0.005	—
*τ*_*c*_	Glutamate clearance time constant	2–100	25	ms
*ζ*	Efficacy of synaptic transmission	0-1	0.75	—

Astrocyte GPCR kinetics				
*O*_*A*_	Agonist binding rate		0.3	*μ*M^−1^s^−1^
*τ*_*A*_	Agonist unbinding time		0.55	s

IP_3_R kinetics				
*O*_2_	Inact. Ca^2+^ binding rate (with Ca^2+^ act.)	0.04–0.18	0.2	*μ*M^−1^s^−1^
*d*_1_	IP_3_ binding affinity	0.1–0.15	0.13	*μ*M
*d*_2_	Inact. Ca^2+^ binding affinity (Ca^2+^ act.)		1.05	*μ*M
*d*_3_	IP_3_ binding affinity (Ca^2+^ inact.)		0.9434	*μ*M
*d*_5_	Act. Ca^2+^ binding affinity		0.08	*μ*M

Calcium fluxes				
*ϱ*_*A*_	ER-to-cytoplasm volume ratio	0.4–0.7	0.18	—
*C*_*T*_	Total ER Ca^2+^ content	3–5	2	*μ*M
Ω_*L*_	Max. Ca^2+^ leak rate	0.05–0.1	0.1	s^−1^
Ω_*C*_	Max. Ca^2+^ release rate by IP_3_Rs	>6	6	s^−1^
*K*_*P*_	Ca^2+^ affinity of SERCA pumps	0.05–0.1	0.05	*μ*M
*O*_*P*_	Max. Ca^2+^ uptake rate	0.4–1.3	0.9	*μ*M s^−1^

IP_3_ production				
*O*_*β*_	Max. rate of IP_3_ production by PLC*β*	0.05–2	1	*μ*M s^−1^
*K*_*δ*_	Ca^2+^ affinity of PLC*δ*	0.1–1	0.5	*μ*M
*κ*_*δ*_	Inhibiting IP_3_ affinity of PLC*δ*	1–1.5	1	*μ*M
*O*_*δ*_	Max. rate of IP_3_ production by PLC*δ*	<0.8	0.05	*μ*M s^−1^

IP_3_ degradation				
Ω_5P_	Max. rate of IP_3_ degradation by IP-5P	>0.05–0.25	0.1	s^−1^
*K*_*D*_	Ca^2+^ affinity of IP_3_-3K	0.4–0.5	0.5	*μ*M
*K*_3K_	IP_3_ affinity of IP_3_-3K	0.7–1	1	*μ*M
*O*_3K_	Max. rate of IP_3_ degradation by IP_3_-3K	>0.6	4.5	*μ*M s^−1^

Gliotransmitter release and time course				
*C*_*θ*_	Ca^2+^ threshold for exocytosis	0.15–0.8	0.5	*μ*M
*τ*_*G*_	Glutamate recycling time constant	0.003–1.5	1.66	s
*U*_*A*_	Resting glutamate release probability	<0.9	0.6	—
*ϱ*_*e*_	Vesicular versus mixing volume ratio		6.5 · 10^−4^	—
*τ*_*e*_	Glutamate clearance time constant	≤300	200	ms

Presynaptic receptors				
*O*_*P*_	Activation rate	>0.3	1.5	*μ*M^−1^s^−1^
*τ*_*P*_	Inactivation time constant	>30–180	120	s
*ξ*	Gliotransmission type	0-1	s.s.	—

Postsynaptic neuron				
*τ*_*m*_	Membrane time constant	20–70	40	ms
*τ*_*r*_	Refractory period	1–5	2	ms
*E*_*L*_	Resting potential	−78.2–−54.8	−60	mV
*v*_*θ*_	Firing threshold	−55–−51	−55	mV
*v*_*r*_	Reset potential	−58–−53	−57	mV
*v*_*p*_	Peak AP amplitude	29.8–41.2	30	mV
*R*_in_	Input resistance	60–150	s.s.	MΩ

Postsynaptic currents				
*τ*_*N*_^*r*^	EPSC rise time	0.4–0.6	0.5	ms
*τ*_*N*_	EPSC decay time	2.7–11.6	10	ms
*J*_*S*_	Synaptic efficacy		4.3	—
*I*_*S*_	EPSP amplitude	0.5–7.5	2	mV

Slow inward currents				
*τ*_*S*_^*r*^	SIC rise time	20–70	20	ms
*τ*_*S*_	SIC decay time	100–800	600	ms
*J*_*A*_	SIC efficacy		68	—
*I*_*A*_	SIC amplitude	1–10	4.5	mV

Spike-timing-dependent plasticity				
*C*_pre_	NMDAR-mediated Ca^2+^ increase per AP		1.0	—
*τ*_pre_^*r*^	NMDAR Ca^2+^ rise time		10	ms
*τ*_pre_	NMDAR Ca^2+^ decay time		30	ms
*W*_*N*_	Synaptic weight		39.7	—
*C*_post_	VDCC-mediated Ca^2+^ increase per AP		2.5	—
*τ*_post_^*r*^	VDCC Ca^2+^ rise time		2	ms
*τ*_post_	VDCC Ca^2+^ decay time		12	ms
*C*_sic_	SIC-mediated Ca^2+^ increase per AP		1.0	—
*τ*_sic_^*r*^	SIC Ca^2+^ rise time		5	ms
*τ*_sic_	SIC Ca^2+^ decay time		100	ms
*W*_*A*_	SIC weight		10.6	—
*η*	Amplification of NMDAR-mediated Ca^2+^		4	—
*θ*_*d*_	LTD threshold		1.0	—
*θ*_*p*_	LTP threshold		2.2	—
*γ*_*d*_	LTD learning rate		0.57	s^−1^
*γ*_*p*_	LTP learning rate		2.32	s^−1^
*ρ*_⋆_	Boundary between UP/DOWN states		0.5	—
*τ*_*ρ*_	Decay time of synaptic change		1.5	s
*σ*	Noise amplitude		0.1	—
*β*	Fraction of synapses in the DOWN state		0.5	—
*b*	UP/DOWN Synaptic strength ratio		4	—

## Appendix

### A. Modeling Methods

#### A.1. Synapse Model with Glutamatergic Gliotransmission

##### A.1.1. Synaptic Release

To study modulation of short-term synaptic plasticity by gliotransmitter-bound extrasynaptically located presynaptic receptors we extend the model originally introduced by De Pittà et al. [73] for astrocyte-mediated heterosynaptic modulation of synaptic release to also account for the homosynaptic scenario. For the sake of clarity, in the following we will limit our description to excitatory (glutamatergic) synapses. Accordingly, synaptic glutamate release is described following Tsodyks [55], whereby upon arrival of an action potential (AP) at time *t*_*k*_, the probability of glutamate resources to be available for release (*u*_*S*_) increases by a factor *u*_0_, while the readily releasable glutamate resources (*x*_*S*_) decrease by a fraction *r*_*S*_(*t*_*k*_) = *u*_*S*_(*t*_*k*_^+^)*x*_*S*_(*t*_*k*_^−^), corresponding to the fraction of effectively released glutamate. In between APs, glutamate resources are reintegrated at a rate of 1/*τ*_*d*_ while *u*_*S*_ decays to zero at a rate of 1/*τ*_*f*_. The equations for *u*_*S*_, *x*_*S*_ thus read [55]

(A.1) τfddtuS=−uS+∑ku01−uSδt−tkτf,τdddtxS=1−xS−∑krStδt−tkτd.

The parameter *u*_0_ in the above equations may be interpreted as the synaptic release probability at rest. Indeed, the synapse is “at rest” when *u*_*S*_ → 0 and *x*_*S*_ → 1, which occurs when the period of incoming Aps is much larger than the synaptic time scales *τ*_*d*_, *τ*_*f*_. Then, in this case, the probability of glutamate release from the synapse by an AP equals *u*_0_.

##### A.1.2. Neurotransmitter Time Course

Assuming a total vesicular glutamate concentration of *Y*_*T*_, the released glutamate, expressed as concentration in the synaptic cleft, is then equal to *Y*_rel_(*t*_*k*_) = *ϱ*_*c*_ *Y*_*T*_ *r*_*S*_(*t*_*k*_), where *ϱ*_*c*_ represents the ratio between vesicular and synaptic cleft volumes. The time course of synaptically released glutamate in the cleft (*Y*_*S*_) depends on several mechanisms, including clearance by diffusion, uptake, and/or degradation [56, 57]. In the simplest approximation, the contribution of these mechanisms to glutamate time course in the cleft may be modeled by a first-order degradation reaction of characteristic time *τ*_*c*_ [58] so that

(A.2) $$\begin{array}{c} \tau_{c}\frac{d}{dt}Y_{S}=-Y_{S}+\underset{k}{\sum }Y_{rel}\delta \left(\;t-t_{k}\;\right)\tau_{c}. \end{array}$$

##### A.1.3. Astrocytic Calcium Dynamics

We assume that only a fraction *ζ* of released glutamate binds to postsynaptic receptors, while the remainder 1 − *ζ* fraction spills out of the cleft and activates astrocytic metabotropic receptors which trigger astrocytic Ca^2+^ signaling. The latter is modeled following Wallach et al. [136] and results from the interplay of four quantities: (i) the fraction of activated astrocytic receptors (*γ*_*A*_); (ii) the cytosolic IP_3_ (*I*) and (iii) Ca^2+^ concentrations (*C*) in the astrocyte; and (iv) the fraction of deinactivated IP_3_ receptors (IP_3_Rs) on the membrane of the astrocyte's endoplasmic reticulum (ER) that mediate Ca^2+^-induced Ca^2+^-release (*h*). In particular, considering each quantity separately, the fraction of astrocytic receptors bound by synaptic glutamate may be approximated, at first instance, by a first-order binding reaction and thus evolves according to [136]

(A.3) $$\begin{array}{c} \tau_{A}\frac{d}{dt}\gamma_{A}=-\gamma_{A}+O_{M}\left(\;1-\zeta \;\right)Y_{S}\left(\;1-\gamma_{A}\;\right)\tau_{A}, \end{array}$$

with *τ*_*A*_ representing the characteristic receptor deactivation (unbinding) time constant. Cytosolic IP_3_ results instead from the complex Ca^2+^-modulated interplay of phospholipase C*β*- and C*δ*-mediated production and degradation by IP_3_ 3-kinase (3K) and inositol polyphosphatase 5-phosphatase (5P) [137–140] and evolves according to the mass balance equation [141]:

(A.4) $$\begin{array}{c} \frac{d}{dt}I=J_{\beta }\left(\;\gamma_{A}\;\right)+J_{\delta }\left(\;C,I\;\right)-J_{3K}\left(\;C,I\;\right)-J_{5P}\left(\;I\;\right), \end{array}$$

where

(A.5) JβγA=OβγA,JδC,I=Oδκδκδ+IHC2,Kδ,J3KC=O3KHC4,KDHI,K3,J5PI=Ω5PI

and *ℋ*(*x* ^*n*^, *K*) denotes the sigmoid (Hill) function *x* ^*n*^/(*x* ^*n*^ + *K* ^*n*^). Finally, cytosolic Ca^2+^ and the IP_3_R gating are described by a set of Hodgkin-Huxley-like equations according to the model originally introduced by Li and Rinzel [142]:

(A.6) ddtC=JCC,h,I+JLC−JPC,ddth=h∞C,I−hτhC,I,

where *J*_*C*_, *J*_*L*_, and *J*_*P*_, respectively, denote the IP_3_R-mediated Ca^2+^-induced Ca^2+^-release from the ER (*J*_*C*_), the Ca^2+^ leak from the ER (*J*_*L*_), and the Ca^2+^ uptake from the cytosol back to the ER by serca-ER Ca^2+^/ATPase pumps (*J*_*P*_) [141]. These terms, together with the IP_3_R deinactivation time constant (*τ*_*h*_) and steady-state probability (*h*_*∞*_), are given by [142, 143]

(A.7) JCC,h,I=ΩCm∞3h3CT−1+ϱAC,m∞C,I=HI,d1HC,d5,JLC=ΩLCT−1+ϱAC,JPC=OPHC2,KP,h∞C,I=d2I+d1d2I+d1+I+d3C,τhC,I=I+d3Ω2I+d1+O2I+d3C.

A detailed explanation of the parameters of the astrocyte model may be found in Table 1.

##### A.1.4. Calcium-Dependent Glutamatergic Gliotransmission

Astrocytic glutamate exocytosis is modeled akin to synaptic glutamate release, assuming that a fraction *x*_*A*_(*t*) of gliotransmitter resources is available for release at any time *t*. Then, every time *t*_*j*_ that astrocytic Ca^2+^ increases beyond a threshold concentration *C*_*θ*_, a fraction of readily releasable astrocytic glutamate resources, that is, *r*_*A*_(*t*_*j*_) = *U*_*A*_ *x*_*A*_(*t*_*j*_^−^), is released into the periastrocytic space and later reintegrated at rate 1/*τ*_*A*_. Hence, *x*_*A*_(*t*) evolves according to [73]

(A.8) $$\begin{array}{c} \tau_{G}\frac{d}{dt}x_{A}=1-x_{A}-\underset{j}{\sum }r_{A}\left(\;t\;\right)\delta \left(\;t-t_{j}\;\right)\tau_{G}. \end{array}$$

Similarly to (A.2), we may estimate the contribution to glutamate concentration in the periastrocytic space (*G*_*A*_), resulting from a quantal glutamate release event by the astrocyte at *t* = *t*_*j*_, as *G*_rel_(*t*_*j*_) = *ϱ*_*e*_ *G*_*T*_ *r*_*A*_(*t*_*j*_), where *G*_*T*_ represents the total vesicular glutamate concentration in the astrocyte and *ϱ*_*e*_ is the volume ratio between glutamate-containing astrocytic vesicles and periastrocytic space. Then, assuming a clearance rate of glutamate of 1/*τ*_*e*_, the time course of astrocyte-derived glutamate in the extracellular space comprised between the astrocyte and the surrounding synaptic terminals is given by

(A.9) $$\begin{array}{c} \tau_{e}\frac{d}{dt}G_{A}=-G_{A}+\underset{j}{\sum }G_{rel}\left(\;t\;\right)\delta \left(\;t-t_{j}\;\right)\tau_{e}. \end{array}$$

##### A.1.5. Presynaptic Pathway for Glutamatergic Gliotransmission

The extracellular glutamate concentration sets the fraction of bound extrasynaptically located presynaptic receptors (*γ*_*S*_) according to [73]

(A.10) $$\begin{array}{c} \tau_{P}\frac{d}{dt}\gamma_{S}=-\gamma_{S}+O_{P}\left(\;1-\gamma_{S}\;\right)G_{A}\tau_{P}, \end{array}$$

where *O*_*P*_ and *τ*_*P*_, respectively, denote the rise rate and the decay time of the effect of gliotransmission on synaptic glutamate release. It is then assumed that modulations of synaptic release by gliotransmitter-bound presynaptic receptors are brought forth by modulations of the resting synaptic release probability; that is, *u*_0_ = *u*_0_(*γ*_*S*_). In an attempt to consider a mechanism as general as possible, rather than focusing on a specific functional dependence for *u*_0_(*γ*_*S*_), we consider only the first-order expansion of this latter [73]; that is,

(A.11) $$\begin{array}{c} u_{0}\left(\;\gamma_{S}\;\right)\approx U_{0}+\left(\;\xi -U_{0}\;\right)\gamma_{S}, \end{array}$$

where *U*_0_ denotes the synaptic release probability at rest in the absence of gliotransmission, whereas the parameter *ξ* lumps, in a phenomenological way, the information on the effect of gliotransmission on synaptic release. For 0 ≤ *ξ* < *U*_0_, *u*_0_ decreases with *γ*_*S*_, consistent with a “release-decreasing” effect of astrocytic glutamate on synaptic release. This could be the case, for example, of astrocytic glutamate binding to presynaptic kinate receptors or group II/III mGluRs [13, 14, 71]. For *U*_0_ < *ξ* ≤ 1, *u*_0_ increases with *γ*_*S*_, consistent with a “release-increasing” effect of astrocytic gliotransmitter on synaptic release, as in the case of presynaptic NMDARs or group I mGluRs [2, 7–9, 11, 12, 72]. Finally, the special case where *ξ* = *U*_0_ corresponds to occlusion, that is, coexistence and balance between release-decreasing and release-increasing glutamatergic gliotransmission at the same synapse resulting in no net effect of this latter on synaptic release.

Although based on glutamatergic synapses, the set of (A.1)–(A.11) provides a general description for modulations of synaptic release mediated by glutamatergic gliotransmission that could also be easily extended to other types of excitatory synapses [144] as well as inhibitory synapses [14, 45, 145, 146].

##### A.1.6. Postsynaptic Pathways for Glutamatergic Gliotransmission: Slow Inward Currents

Postsynaptic astrocyte-mediated slow inward currents take place through extrasynaptic NMDA receptors. The subunit composition of these receptors however remains unclear [77]. Several studies reported SICs to be inhibited by antagonists of NR2B-containing NMDARs [21, 24, 78]; however there is also evidence that other NMDAR types could be involved, possibly containing subunits NR2C or NR2D [25]. Being mediated by NMDA receptors, SICs are likely affected by voltage dependence of the Mg^2+^ block of these receptors. Although there is evidence that SICs rate and frequency could indeed depend on extracellular Mg^2+^ [21], the effective nature of the possible voltage dependence of SICs has not been elucidated. Moreover, the potential diversity of subunit composition of receptors mediated SICs could also result in different voltage dependencies, strong for NR2B-containing receptors, akin to postsynaptic NMDARs, and weak otherwise [111]. Accordingly, in this study we neglect the possible voltage dependence of SICs arguing that this is not substantially changing the essence of our results (see Discussion). In this fashion, denoting postsynaptic SICs by *i*_*A*_(*t*), we model them by a difference of exponentials according to

(A.12) τSrddtiAt=−iAt+I^ABAtτSr,

(A.13) τSddtBAt=−BAt+J^AGAtτS,

where *τ*_*S*_^*r*^ and *τ*_*S*_, respectively, are rise and decay time constants for SICs. The two scaling factors ${\hat{I}}_{A}$ and ${\hat{J}}_{A}$ are taken such that the SIC maximum in correspondence with an event of glutamate release by the astrocyte is equal to a constant value *I*_*A*_ (see Appendix B).

##### A.1.7. Postsynaptic Neuron

Postsynaptic action potential firing is modeled by a leaky integrate-and-fire (LIF) neuron [91, 92]. Accordingly, the membrane potential (*v*) of the postsynaptic neuron evolves as

(A.14) $$\begin{array}{c} \tau_{m}\frac{d}{dt}v=E_{L}-v+i_{S}\left(\;t\;\right)+i_{A}\left(\;t\;\right), \end{array}$$

where *τ*_*m*_ denotes the membrane time constant and *i*_*S*_(*t*) represents the excitatory synaptic input to the neuron. Every time *v* crosses the firing threshold *v*_*θ*_, an AP is emitted and the membrane potential is reset to *v*_*r*_ and kept at this value for the duration of a refractory period *τ*_*r*_.

For synaptic currents, we only consider the AMPA receptor-mediated fast component of EPSCs. Accordingly, we consider two possible descriptions for *i*_*S*_(*t*). In Figures 3 and 4, we assume that the rise time of synaptic currents is very short compared to the relaxation time of these latter [147–149], so that *i*_*S*_(*t*) can be modeled by a sum of instantaneous jumps of amplitude ${\hat{J}}_{S}$, each followed by an exponential decay [91]; that is,

(A.15) $$\begin{array}{c} \tau_{N}\frac{d}{dt}i_{S}\left(\;t\;\right)=-i_{S}\left(\;t\;\right)+{\hat{J}}_{S}\zeta Y_{S}\left(\;t\;\right)\tau_{N}. \end{array}$$

In the presence of gliotransmitter-mediated slow inward currents instead (Figure 5), we model synaptic currents similarly to these latter; that is,

(A.16) τNrddtiSt=−iSt+I^SBStτNr,

(A.17) τNddtBSt=−BSt+J^SζYStτN,

where *τ*_*N*_^*r*^, *τ*_*N*_, respectively, denote EPSC rise and decay time constants; the scaling factor ${\hat{J}}_{S}$ is taken such that synaptic releases result in unitary increases of the gating variable *B*_*S*_, and similarly, ${\hat{I}}_{S}$ is set such that an increase in synaptic current ensuing from quantal synaptic release equals to *I*_*S*_.

#### A.2. Spike-Timing-Dependent Plasticity

##### A.2.1. Postsynaptic Calcium Dynamics

Spike-timing-dependent plasticity is modeled by the nonlinear calcium model introduced by Graupner and Brunel [102], which was modified to include short-term synaptic plasticity as well as astrocyte-mediated SIC contribution to postsynaptic Ca^2+^. Accordingly, postsynaptic Ca^2+^ dynamics, *c*(*t*), result from the sum of three contributions: (i) Ca^2+^ transients mediated by NMDA receptors, activated by synaptic glutamate whose release probability from the presynaptic bouton may be modulated by gliotransmission (*c*_pre_), (ii) Ca^2+^ transients mediated by gliotransmitter-triggered NMDA-mediated SICs (*c*_sic_), and (iii) Ca^2+^ transients due to activation of voltage-dependent Ca^2+^ channels (VDCCs) by postsynaptic backpropagating APs (*c*_post_). All three Ca^2+^ transients are accounted for by a difference of exponentials. In particular, presynaptic Ca^2+^ transients are described by

(A.18) τprerddtcpre=−cpre+C^preRpreτprer,τpreddtRpre=−Rpre+WNζYSτpre,

where *τ*_pre_^*r*^, *τ*_pre_ are rise and decay time constants of the Ca^2+^ transient; ${\hat{C}}_{pre}$ is a normalization constant such that the maximal amplitude of the transient is *C*_pre_; *W*_*N*_ is the “weight” of presynaptic Ca^2+^ transients triggered by synaptic glutamate (*Y*_*S*_, equation (A.2)).

Similarly to presynaptic ones, calcium transients due to SICs mediated by gliotransmission are given by

(A.19) τsicrddtcsic=−csic+C^sicRsicτsicr,τsicddtRsic=−Rsic+WAGAτsic,

where *τ*_sic_^*r*^ and *τ*_sic_^*d*^ are the rise and decay time constants of the Ca^2+^ transient; and ${\hat{C}}_{sic}$ is a normalization constant such that the maximal amplitude of the transient is *C*_sic_; *W*_*A*_ is the “weight” of SIC-mediated Ca^2+^ transients triggered by perisynaptic gliotransmitter (*G*_*A*_, equation (A.9)).

Finally, postsynaptic Ca^2+^ transients caused by bAPs are modeled by [102]

(A.20) τpostrddtcpost=−cpost+C^postRpostτpostr,τpostddtRpost=−Rpost+1+ηcpre∑iδt−tiτpost,

where the sum goes over all postsynaptic APs occurring at times *t*_*i*_; *τ*_post_^*r*^ and *τ*_post_ are the rise and decay time constants of the Ca^2+^ transient; ${\hat{C}}_{post}$ is a scaling factor such that the maximal amplitude of the transient is *C*_post_, and the parameter *η* denotes how much a bAP-evoked Ca^2+^ transient is increased by preceding presynaptic stimulation.

##### A.2.2. Synaptic Efficacy

The temporal evolution of synaptic efficacy *ρ*(*t*) depends on postsynaptic Ca^2+^ dynamics *c*(*t*) = *c*_pre_(*t*) + *c*_sic_(*t*) + *c*_post_(*t*) and is described by the first-order differential equation [102]:

(A.21) τρddtρ=−ρ1−ρρ⋆−ρ+γp1−ρΘct−θp−γdρΘct−θd+Noiset,

where *ρ*_⋆_ is the boundary of the basins of attraction of UP and DOWN states of synaptic efficacy, that is, the states for which *ρ* = 1 and *ρ* = 0, respectively; *θ*_*d*_, *θ*_*p*_ denote the depression (LTD) and potentiation (LTP) thresholds, and *γ*_*d*_, *γ*_*p*_ measure the corresponding rates of synaptic decrease and increase when these thresholds are exceeded; Θ(·) denotes the Heaviside function, that is, Θ(*c* − *θ*) = 0 for *c* < *θ* and Θ(*c* − *θ*) = 1 for *c* ≥ *θ*. The last term lumps an activity-dependent noise term in the form of $Noise(t)=\sigma \sqrt{\tau_{\rho }}\sqrt{\Theta \left(c(t)-\theta_{d}\right)+\Theta \left(c(t)-\theta_{p}\right)}\cdot \varpi (t)$, where *ϖ*(*t*) is a Gaussian white noise process with unit variance density. This term accounts for activity-dependent fluctuations stemming from stochastic neurotransmitter release, stochastic channel opening, and diffusion [102].

#### A.3. STDP Curves

To construct the STDP curves of Figures 6–8, we follow the rationale originally described by Graupner and Brunel [102] and consider the average synaptic strength of a synaptic population after a stimulation protocol of *n* pre-post (or post-pre) pairs presented at time interval *T*. With this aim, synaptic strength is surmised to be linearly related to *ρ* as *w* = *w*_0_ + *ρ*(*w*_1_ − *w*_0_), where *w*_0_ and *w*_1_ are the synaptic strength of the DOWN/UP states for which *ρ*_0_, that is, the initial value of *ρ* at *t* = 0, is 0 or 1, respectively. In this fashion, *w* may be thought of as a rescaled version of equivalent experimental measures of synaptic strength such as the excitatory postsynaptic potential (EPSP) or excitatory postsynaptic current (EPSC) amplitude, the initial EPSP slope, or the current in 2 ms windows at the EPSC peak. Accordingly, before a stimulus protocol, a fraction *β* of the synapses is taken in the DOWN state, so that the average initial synaptic strength is ${\bar{w}}_{0}=\beta w_{0}+(1-\beta )w_{1}$. Then, after the stimulation protocol, the ensuing average synaptic strength is ${\bar{w}}_{1}=w_{0}((1-𝒰)\beta +𝒟(1-\beta ))+w_{1}(𝒰\beta +(1-𝒟)(1-\beta ))$, where *𝒰* and *𝒟* represent the UP and DOWN transition probabilities, respectively. As in experiments, we consider the change in synaptic strength as the ratio between the average synaptic strengths after and before the stimulation; that is,

(A.22) Δρ=1−Uβ+D1−β+bUβ+1−D1−ββ+1−βb,

with *b* = *w*_1_/*w*_0_. Under the hypotheses of this study, that is, *T* ≪ *τ*_*ρ*_ and *γ*_*d*_, *γ*_*p*_ are large, the transition probabilities *𝒰* and *𝒟* may be analytically solved and read [102]:

(A.23) Uρ0=121+erf−ρ⋆−ρ¯+ρ−ρ0e−nT/τσρ1−e−2nT/τ,Dρ0=121−erf−ρ⋆−ρ¯+ρ−ρ0e−nT/τσρ1−e−2nT/τ,

where erf denotes the standard error function, defined as $erf(x)=\left(2/\sqrt{\pi }\right)\int_{0}^{x}e^{-t^{2}}dt$ and

(A.24) ρ¯=ΓpΓd+Γp,σρ2=αd+αpΓd+Γpσ2,τ=τρΓd+Γp,

with Γ_*i*_ = *γ*_*i*_ *α*_*i*_ and *α*_*i*_ = (1/*nT*)∫_0_^*nT*^Θ(*c*(*t*) − *θ*_*i*_)d*t* with *i* = *d*, *p*.

#### A.4. Simulations

The model was implemented in Brian 2.0 [150]. Simulations and data analysis were serendipitously designed and performed by the open source programming language Python 2.7 [151]. The code is available online and can be downloaded from terminal by “git clone https://mauriziodepitta@bitbucket.org/mauriziodepitta/public.code.git -b NeuralPlasticity-2016.”

### B. Parameter Estimation

#### B.1. Synaptic Parameters

Glutamate release probability *U*_0_ of central excitatory synapses is generally comprised between ~0.09 [152] and ~0.6–0.9 [153, 154], with lower values mostly consistent with facilitating synapses [155]. Facilitation time constants *τ*_*f*_ may be estimated by the decay time of intracellular Ca^2+^ increases at presynaptic terminals upon arrival of action potentials [156, 157]. With this regard, typical decay times for Ca^2+^ transients are reported to be <500 ms [157], with an upper bound within 0.65–2 s [156]. Concerning depression time constants instead, experiments have reported glutamate-containing vesicles in the readily releasable pool to preferentially undergo rapid endocytosis within 1-2 s after release [158], although vesicle recycling could also be as fast as 10–20 ms [153, 159].

Estimates in hippocampal synapses suggest that the readily releasable pool could contain between 2 and 27 vesicles [152] which are essentially spherical with average outer diameter *d*_*S*_ equal to 39.2 ± 11.4 nm and in the range of 23–49 nm [160, 161]. Subtracting to this value a 6 nm thick vesicular membrane [152], the inner diameter of a vesicle can then be estimated within 16–38 nm, corresponding to a mean vesicular volume Λ_*S*_ in the range of 2.1 · 10^−21^–28.7 · 10^−21^ dm^3^. Given that vesicular glutamate concentration is reported in the range of 60–210 mM [160–162], then, considering a pool of 10 vesicles with average diameter of 30 nm (i.e., average volume Λ_*S*_ ≈ 14.1 · 10^−21^ dm^3^) and average vesicular neurotransmitter concentration of 60–100 mM [161], the total neurotransmitter vesicular content ranges up to *Y*_*T*_ = (10)(60–100 mM) = 300–1000 mM. Assuming a typical neurotransmitter release time of *t*_rel_ = 25 *μ*s [163] and a diffusion constant for glutamate in the synaptic cleft of *D*_Glu_ = 0.33 *μ*m^2^/ms [164], the average diffusion length (*ℓ*_*c*_) of a glutamate molecule from the release site can be estimated by the Einstein-Smoluchowski relationship [152] whereby *ℓ*_*c*_ = √(2 · *D*_Glu_ · *t*_rel_) = √(2 · 0.33 *μ*m^2^/ms · 0.025 ms) ≈ 0.129 *μ*m. Thus, the associated mixing volume Λ_*c*_, namely, the effective diffusion volume which the released glutamate has rapid access to, can be estimated by the volume of the disk of radius *ℓ*_*c*_ and thickness *h*_*c*_ equal to the average width of the synaptic cleft [165]. Considering *h*_*c*_ = 20 nm [152], it is Λ_*c*_ = *πℓ*_*c*_^2^ *h*_*c*_ = *π* · (0.129 *μ*m)^2^(0.020 *μ*m) ≈8.89 · 10^−18^ dm^3^ which falls in the experimental range of volumes of nonsynaptic interfaces at hippocampal synapses elsewhere reported [166]. Considering vesicular release from at least 3 independent sites [167], it follows that the ratio between vesicle volume and mixing volume is *ϱ*_*c*_ = Λ_*S*_/Λ_*c*_ = (3)(14.1 · 10^−21^ dm^3^)/(8.89 · 10^−18^ dm^3^) ≈ 0.005, so that the contribution to the concentration of glutamate in the extracellular space following a release event is *Y*_rel_ = *ϱ*_*c*_ · *U*_0_ · *Y*_*T*_. Hence, for a sample value of *U*_0_ = 0.5 [153] with a choice of *Y*_*T*_ = 500 mM, for example, the latter equals *Y*_rel_ = (0.005)(0.5)(500 mM) ≈ 1.25 mM.

Such released glutamate is then rapidly cleared from the extracellular space by combined action of diffusion and uptake by transporters [168]. As a result the time course of glutamate in the synaptic cleft is short, with an estimated decay constant within ~2–10 ms [57, 169]. However, slower clearance times could also be possible since resting glutamate concentrations in the extracellular space surrounding activated synapses are recovered only ~100 ms after the stimulus [170, 171]. Based on these considerations, we consider an intermediate value of glutamate clearance time of *τ*_*c*_ = 25 ms.

#### B.2. Astrocyte Parameters

Astrocyte parameters reported in Appendix C were estimated on extensive numerical explorations of the astrocyte model aimed at reproducing experimental whole-cell Ca^2+^ elevations with rise and decay time constants, respectively, in the ranges of 3–20 s and 3–25 s and with full-width half-maximum (FWHM) values within 5–160 s [172–174]. In doing so, we considered a ratio between ER and cytoplasmic volumes (*ϱ*_*A*_) of 0.18 in line with the experimental observation that the probability of ER localization in the cytoplasmic space at astrocytic somata is within ~20–70% [175]. Moreover, the cell's total Ca^2+^ concentration (measured with respect to the cytoplasmic volume) was fixed at *C*_*T*_ = 2 *μ*M, while Ca^2+^ affinity of sarco-ER pumps (SERCAs) was taken equal to 0.05 *μ*M [176, 177], assuring peak Ca^2+^ concentrations < 5 *μ*M in agreement with experiments [178, 179].

Activation rate and unbinding time constants *O*_*A*_, *τ*_*A*_ of astrocytic receptors may be estimated by rise times of agonist-triggered Ca^2+^ signals. With this regard, application of 50 *μ*M of DHPG, a potent agonist of group I subtype 5 mGluRs, the main type of mGluRs expressed by astrocytes [180], triggered submembrane Ca^2+^ signals characterized by a rise time *τ*_*r*_ = 0.272 ± 0.095 s. Because mGluR5 affinity (*K*_0.5_) for DHPG is ~2 *μ*M [181], which is much smaller than the applied agonist concentration, receptor saturation may be assumed in those experiments so that the receptor activation rate by DHPG (*O*_DHPG_) can be expressed as a function of *τ*_*r*_ [165] whereby *O*_DHPG_ ≈ *τ*_*r*_(50 *μ*M)^−1^ = 0.055–0.113 *μ*M^−1^ s^−1^ and, accordingly, *Ω*_DHPG_ = 1/*O*_DHPG_ *K*_0.5_ ≈ 4–10 s. Corresponding rate constants for glutamate may then be estimated by the latter, assuming similar kinetics yet with *K*_0.5_ = *K*_*A*_ = 1/*O*_*A*_ *τ*_*A*_ ≈ 3–10 *μ*M [182], that is, 1.5–5-fold larger than *K*_0.5_ for DHPG. Moreover, since rise times of Ca^2+^ signals triggered by nonsaturating physiological stimuli are somehow faster than in the case of DHPG [10], it may be assumed that *O*_*N*_ > *O*_DHPG_. With this regard, for a choice of *O*_*A*_ ≈ 3*O*_DHPG_ = 0.3 *μ*M^−1^ s^−1^, with *K*_*A*_ = 6 *μ*M such that *τ*_*A*_ = 1/(0.3 *μ*M^−1^s^−1^)/(6 *μ*M) = 0.55 s, a peak synaptic glutamate concentration of *Y*_rel_ = 1200 *μ*M, with *τ*_*c*_ = 25 ms, results in a maximum average fraction of bound receptors of ~0.75–0.9 that occurs within ~70 ms from synaptic release, in good agreement with experimentally reported rise times.

#### B.3. Gliotransmission

Exocytosis of glutamate from astrocytes is reported to occur by Ca^2+^ concentrations increasing beyond a threshold value *C*_*θ*_ ≈ 0.15–0.8 *μ*M. In this study we specifically consider *C*_*θ*_ = 0.5 *μ*M. Glutamate-containing vesicles found in astrocytic processes have regular (spherical) shape with typical diameters (*d*_*A*_) within ~20–110 nm [63, 183]. The corresponding vesicular volume Λ_*A*_ then is within ~2–700·10^−21^ dm^3^. Vesicular glutamate content is approximately the same, or at least as low as one-third of synaptic vesicles in adjacent nerve terminals [8, 161, 184]. Thus, considering a range of synaptic vesicular glutamate content within ~60–150 mM [162], astrocytic vesicular glutamate concentration (*G*_*v*_) is likely within ~20–150 mM [161].

The majority of glutamate vesicles in astrocytic processes clusters in close proximity (i.e., <100 nm) to the plasma membrane, but about half of them are found within a distance of 40–60 nm from the ventral side of the membrane, suggesting existence of “docked” vesicles in astrocytic processes akin to synaptic terminals [8, 161]. Borrowing the synaptic rationale whereby docked vesicles approximately correspond to readily releasable ones [152], then the average number of astrocytic glutamate vesicles available for release (*n*_*A*_) could be within ~1–6 [8]. Hence, the total vesicular glutamate releasable by an astrocyte may be estimated within *G*_*T*_ = *n*_*A*_ *G*_*A*_ = 20–900 mM.

Astrocytic vesicle recycling (*τ*_*G*_) likely depends on the mode of exocytosis. Both full-fusion of vesicles and kiss-and-run events have been observed in astrocytic processes [63] with the latter seemingly occurring more often [63, 185]. The fastest recycling pathway corresponds to kiss-and-run fusion, where the rate is mainly limited by vesicle fusion with plasma membrane and subsequent pore opening [186]. Indeed, reported pore-opening times in this case can be as short as 2.0 ± 0.3 ms [185]. The actual recycling time however could be considerably longer if we take into account that, even for fast release events confined within 100 nm from the astrocyte plasma membrane, vesicle reacidification could last ~1.5 s [187].

Considering a value for the diffusion constant of glutamate in the extracellular space of *D*_Glu_ = 0.2 *μ*m^2^/ms [164] and a vesicle release time *t*_rel_ ≈ 1 ms [185], the average diffusion distance travelled by astrocytic glutamate molecules into the extracellular space is [152] *ℓ*_*e*_ = √(2 · 0.2 *μ*m^2^/ms · 1 ms) ≈ 0.63 *μ*m. Then, assuming that the mixing volume of released glutamate Λ_*e*_ in the extracellular space coincides with one-tenth (i.e., 0.1) of the ideal diffusion volume in free space [188], it is Λ_*e*_ = (0.1)4*πℓ*_*e*_^3^/3 ≈ 10^−16^ dm^3^. Accordingly, considering an average vesicular diameter *d*_*A*_ = 50 nm, so that Λ_*A*_ ≈ 65 · 10^−21^ dm^3^, the ratio *ϱ*_*e*_ between vesicular and mixing volumes for astrocytic glutamate diffusion can be estimated to be of the order of *ϱ*_*e*_ = Λ_*A*_/Λ_*e*_ = (65 · 10^−21^ dm^3^)/10^−16^ dm^3^ ≈ 6.5 · 10^−4^. For an astrocytic pool of releasable glutamate of *G*_*T*_ = 200 mM and a release probability *U*_*A*_ = 0.6, it follows that the extracellular peak glutamate concentration after exocytosis is ${\hat{G}}_{A}=(6.5\cdot 10^{-4})(0.6)(200\;mM)\approx 78$ *μ*M, in agreement with experimental measurements [189]. Finally, imaging of extrasynaptic glutamate dynamics in hippocampal slices hints that glutamate clearance is fast and mainly carried out within <300 ms of exocytosis [171]. Therefore, we consider a characteristic clearance time constant for glutamate in the periastrocytic space of *τ*_*e*_ = 200 ms.

#### B.4. Presynaptic Receptors

We set the activation rate and inactivation time constants of presynaptic receptors, that is, *O*_*P*_ and *τ*_*P*_, to reproduce the experimentally reported rapid onset of the modulatory effect on synaptic release exerted by those receptors, namely, within 1–5 s from glutamate exocytosis by the astrocyte and the slow decay of this modulation, which is of the order of tens of seconds at least [7, 8]. In particular, inhibition of synaptic release following activation of presynaptic mGluRs by astrocytic glutamate could last from tens of seconds [71] to ~2–3 min [13]. Similarly, group I mGluR-mediated enhancement of synaptic release following a single Ca^2+^ elevation in an astrocyte process may last as long as ~30–60 s [2, 7]. Values within ~1-2 min however have also been reported in the case of an involvement of NMDARs [8, 190]. No specific assumption is made on the possible ensuing peak of receptor activation by a single glutamate release by the astrocyte.

#### B.5. Postsynaptic Neuron

The membrane time constant of pyramidal neurons is typically within 20–70 ms [191, 192] in correspondence with a membrane potential at rest in the range of −66.5 ± 11.7 mV [147, 148, 192–196]. Firing threshold (*v*_*θ*_) values are on average around −53 ± 2 mV [195, 197, 198], resulting in after-spike reset potentials (*v*_*r*_) that are 2-3 mV below the firing threshold [192, 195, 199]. The peak of action potentials is artificially set in simulations to *v*_*p*_ = 30 mV [193, 197], whereas the refractory period for neuronal action potential generation is fixed at *τ*_*r*_ = 2 ms [93, 195, 197].

#### B.6. Postsynaptic Currents

AMPA receptor-mediated EPSCs recorded at central synapses are characterized by small rise time constants (*τ*_*N*_^*r*^), namely, within 0.2–0.6 ms [147–149, 196], and decay time constants (*τ*_*N*_) in the range of 2.7–11.6 ms [149, 191, 196, 200]. Furthermore, whole-cell recordings of EPSCs for quantal-like glutamatergic stimulation report amplitudes for these currents generally within ~15.5–30 pA [191, 196], and similarly, corresponding somatic depolarizations (EPSPs) are in a wide range of values comprised within ~0.5–7.2 mV, consistent with large quantal size variability of glutamate release from presynaptic terminals [201]. Accordingly, in the simulations of Figure 5 we set *τ*_*N*_^*r*^ = 0.5 ms and *τ*_*N*_ = 10 ms and take the two scaling factors ${\hat{J}}_{S},{\hat{I}}_{S}$ in (A.16) such that [202]

(B.1) J^S=JSϱcYTτN,I^S=IS1/τN−1/τNrτNr/τNτN/τN−τNr−τNr/τNτNr/τN−τNr.

In this fashion, with 3/4 of released neurotransmitter reaching postsynaptic receptors (i.e., *ζ* = 0.75), setting *J*_*S*_ = 4.27 results in EPSP amplitudes approximately equal to *I*_*S*_. In order to convert PSCs and SICs from voltage to current units, we divide them by typical neuronal input resistance (*R*_in_) values which are generally reported in the range of ~60–150 MΩ [192, 193, 196].

#### B.7. Slow Inward Currents

Astrocyte-mediated SICs are documented in a wide range of amplitudes that spans from >10 pA [11, 21, 22, 26, 32] to >200 pA [21, 25, 85, 87], although their majority is mostly found within 30–80 pA in physiological conditions [21, 26]. SICs kinetics also likely vary depending on subunit composition of SIC-mediating NMDA receptors [203]. In general, assuming NR2B-containing NMDARs as the main receptor type mediating SICs [77], mean SICs rise and decay times are, respectively, reported in ~30–90 ms and ~100–800 [20, 21]. Accordingly, in the simulations of Figure 5 we set *τ*_*S*_^*r*^ = 30 ms and *τ*_*N*_ = 600 ms and take the two scaling factors ${\hat{J}}_{S},{\hat{I}}_{S}$ in (A.12) such that

(B.2) J^A=JAϱeGTτS,I^A=IA1/τS−1/τSrτSr/τSτN/τN−τSr−τSr/τSτSr/τS−τSr.

In this fashion, we ultimately choose the value of *J*_*S*_ to account for SIC-mediated depolarizations approximately equal to *I*_*A*_. As SIC amplitudes are generally reported in terms of current rather than voltage units, we estimate somatic depolarizations ensuing from SICs by expressing these latter in terms of typical EPSCs. Thus, for example, for individual EPSCs of 30 pA that generate 2 mV EPSPs, realistic SICs could be regarded on average to be ~1–5-fold these EPSCs and thus contribute to a similar extent to 1–5 times typical EPSPs, that is, ~2–10 mV.

#### B.8. Spike-Timing-Dependent Plasticity

We consider the set of parameters for the nonlinear Ca^2+^ model by Graupner and Brunel [102, Figure S6] originally proposed by these authors to qualitatively reproduce the classic STDP curve [123]. In addition, SIC-mediated postsynaptic Ca^2+^ transients are assumed similar to presynaptically triggered Ca^2+^ transients but with likely longer rise and decay times. Finally, both presynaptically mediated and SIC-mediated Ca^2+^ transients are rescaled by equations analogous to (B.1) and (B.2) in order to obtain transient peak amplitudes equal to *C*_pre_ and *C*_sic_, respectively.

### C. Parameter Ranges and Values

Values of model parameters used in our simulations are summarized in Table 1. Blank table entries are for those parameters whose value was either taken from previously published studies [102, 136, 141] or estimated on the basis of other model parameters (see Appendix B). Simulation specific (s.s.) parameter values are instead specified within figure captions.

## Abbreviations

AMPAR: *α*-Amino-3-hydroxy-5-methyl-4-isoxazolepropionic acid receptor
AP: Action potential
bAP: Back-propagating action potential
ER: Endoplasmic reticulum
GPCR: G protein-coupled receptor
LTD: Long-term depression
LTP: Long-term potentiation
mGluR: Metabotropic glutamate receptor
NMDA(R): N-Methyl-d-aspartate (receptors)
PAR1: Protease-activated receptor 1
PPR: Pair pulse ratio
(E)PSC: (Excitatory) postsynaptic current
(E)PSP: (Excitatory) postsynaptic potential
SIC: Slow inward current
SERCA: Sarco/endoplasmic reticulum Ca^2+^/ATPase
STDP: Spike-timing-dependent plasticity
VDCC: Voltage-dependent calcium channel.
